# Phylogeny of the *Inocybe asterospora* group (*Inocybaceae*, *Agaricales*) uncovers cryptic diversity in warm-temperate and subtropical China

**DOI:** 10.3897/mycokeys.141.210930

**Published:** 2026-10-06

**Authors:** Wen-Jie Yu, Yu-Peng Ge, Wan-Yi Ren, Qin Na, Yan-Ru Xu, Ya-Ping Hu, Yu-Guang Fan

**Affiliations:** 1 Engineering Research Center of Tropical Medicine Innovation and Transformation of Ministry of Education, Hainan Provincial Key Laboratory of Research and Development on Tropical Herbs, School of Pharmaceutical Sciences, Hainan Medical University, Haikou, 571199, China Engineering Research Center of Tropical Medicine Innovation and Transformation of Ministry of Education, Hainan Provincial Key Laboratory of Research and Development on Tropical Herbs, School of Pharmaceutical Sciences, Hainan Medical University Haikou China; 2 School of Public Health, Key Laboratory of Tropical Translational Medicine of Ministry of Education, Hainan Medical University, Haikou, 571199, China School of Public Health, Key Laboratory of Tropical Translational Medicine of Ministry of Education, Hainan Medical University Haikou China; 3 School of Horticulture, Ludong University, Yantai 264025, China School of Horticulture, Ludong University Yantai China; 4 Nanjing Institute of Environmental Sciences, Ministry of Ecology and Environment, State Environmental Protection Scientific Observation and Research Station for Ecological Environment of Wuyi Mountains, Nanjing 210042, China Nanjing Institute of Environmental Sciences, Ministry of Ecology and Environment, State Environmental Protection Scientific Observation and Research Station for Ecological Environment of Wuyi Mountains Nanjing China

**Keywords:** *
Agaricales
*, *
Inocybaceae
*, new taxa, taxonomy

## Abstract

The *Inocybe asterospora* group is a morphologically distinctive lineage with global distribution, yet its taxonomy and phylogenetic relationships in East Asia remain poorly understood. Here, we conducted the first multi-locus phylogenetic assessment of the *I. asterospora* complex from warm-temperate and subtropical China using ITS, LSU, and *rpb2* sequences, combined with morphological observations. Our analyses uncovered three new species, namely *I. taishanensis***sp. nov**., *I. draconoculus***sp. nov**., and *I. pretiosa***sp. nov**., substantially expanding the known diversity of this group in East Asia. The phylogeny recovered four geographically distinct subclades within the *Asterospora* group, although their interrelationships remained statistically unsupported. We further demonstrated that previous Chinese records of “*I. asterospora*” represent a species complex, and that the confirmed distribution of true *I. asterospora* is confined to northern China. Records from North America and southern China lack molecular confirmation and likely represent misidentifications or undescribed taxa. These findings indicate that the species diversity of this group in East Asia has been substantially underestimated.

## Introduction

The genus *Inocybe* (Fr.) Fr. is one of the most species-rich and taxonomically challenging groups of ectomycorrhizal agaricoid fungi, with over 1,100 species recognized worldwide (Aïgnon et al. 2021). Its members associate with a broad range of woody plants, forming obligate symbioses that contribute substantially to temperate and boreal forest ecosystems (Kuyper 1986; Matheny et al. 2020). Many *Inocybe* species produce muscarine and other neurotoxins, and accidental ingestions have resulted in cholinergic poisoning incidents in China and other regions (Lurie et al. 2009; Wu et al. 2019; Li et al. 2020, 2021a, 2022, 2023, 2024, 2025; Xu et al. 2020; Li et al. 2021b). These incidents underscore the practical need for reliable species identification. However, delimiting species in *Inocybe* remains notoriously difficult, owing to pronounced morphological plasticity, subtle interspecific differences, and a general lack of consistent macroscopic diagnostic characters (Kuyper 1986; Esteve-Raventós et al. 2018; Dovana et al. 2023). Recent applications of multi-locus molecular phylogenetics have greatly resolved species boundaries and evolutionary relationships within the genus, uncovering numerous cryptic taxa and previously unrecognized lineages (Horak et al. 2015; He et al. 2022; Matheny et al. 2022; Chen et al. 2024; Gao et al. 2025; Zhu et al. 2025).

The *Inocybe asterospora* group was first recognized by Matheny and Bougher (2017). It is one of the morphologically distinctive groups within *Inocybe*, characterized by nodulose to distinctly stellate basidiospores. The type species, *I. asterospora* Quél., was originally described from France by Lucien Quélet in 1879 based on its unique star-shaped spore ornamentation (Quélet 1879). Historically, this species was placed in the genus *Astrosporina* J. Schröt. proposed by Rea (1922) to accommodate nodulose-spored species; however, subsequent molecular phylogenetic analyses demonstrated that *Astrosporina* was nested within *Inocybe* and thus the genus was synonymized under *Inocybe* (Matheny 2009). *Inocybe asterospora* is a widespread temperate species, with records from Europe, Asia, North America, and parts of Australasia, typically occurring in lowland to montane broad-leaved forests, forming ectomycorrhizal associations with trees such as *Quercus*, *Carpinus*, *Fagus*, and *Castanea* (Horak 1977, 1979; Stangl 1989; Breitenbach and Kränzlin 2000; Kobayashi 2002; Ferrari 2006; Ludwig 2017). The species is also documented from several Chinese provinces spanning warm-temperate to subtropical regions, including Jilin, Liaoning, Inner Mongolia, Beijing, Shaanxi, Sichuan, Yunnan, Zhejiang, Fujian, and Jiangxi (Wang and Bau 2009; Fan and Bau 2010; Li et al. 2016; Bau 2022; Ren et al. 2024).

However, the *Inocybe asterospora* group is by no means a monotypic assemblage. Over the past century, a growing number of morphologically similar species sharing stellate or prominently nodulose spores have been described from various parts of the world. Kauffman (1924) described *I. nodulosa* Kauffman from North America, characterized by a striate pileus with a crenate margin and a distinctly bulbous stipe base. Smith (1941) erected *I. insignis* A.H. Sm. from the Great Smoky Mountains, notable for its greenish bruising reaction, which was later confirmed by molecular data to be closely allied to the *I. asterospora* group (Kuo and Matheny 2015). In Australia and New Zealand, Matheny and Bougher (2017) described two new species, *I. vagata* Matheny & Bougher and *I. nobilissima* Matheny & Bougher, both possessing stellate spores and bulbous stipe bases, with phylogenetic analyses showing them to be sister to the northern hemisphere *I. asterospora* complex. In tropical and subtropical Asia, Horak et al. (2015) described *I. pileosulcata* from Thailand and Malaysia, distinguished by a distinctly sulcate pileus, while Latha and Manimohan (2017) added two additional species from India, *I. rekhankitha* K.P.D. Latha & Manim. and *I. silvana* K.P.D. Latha & Manim., based on combined morphological and molecular evidence. Collectively, these findings suggest that the *I. asterospora* group represents a globally distributed clade with considerable uncovered diversity, particularly in under-explored regions.

To date, at least seven taxa have been recognized as closely related within the *I. asterospora* group. However, no study has specifically addressed the phylogeny of this species group, particularly in East Asia. Most existing records of *I. asterospora* from China are based predominantly on morphological identifications (Du et al. 1980; Xue 1984; Bau 2022), with only a few specimens having been subjected to molecular confirmation. In this study, we conducted a comprehensive morphological and multi-locus phylogenetic investigation of the *I. asterospora* group based on specimens collected from warm-temperate and subtropical China, supplemented by comparative material from other regions. We employed three molecular markers, the internal transcribed spacer (ITS) region of nuclear ribosomal DNA, the nuclear large subunit ribosomal RNA (nLSU), and the second-largest subunit of RNA polymerase II (*rpb2*) to reconstruct the phylogenetic relationships of the group and to assess species boundaries. Our specific objectives were to (1) evaluate the phylogenetic placement of Chinese collections within the *I. asterospora* clade; (2) clarify the species delimitation among morphologically similar taxa; and (3) describe new species based on integrated evidence from phylogeny, morphology, and ecology.

## Materials and methods

### Sampling and morphological examination

The specimens examined in this study originated from Jilin, Shandong, Shaanxi, Ningxia, Hubei of China. Fresh basidiomata were characterized in the field, with color codes referenced to Kornerup and Wanscher (1978). Habitat information, including vegetation type, substrate and accompanying plant species, was meticulously noted for each locality, along with geographic coordinates and elevation captured by a handheld GPS unit. Dehydration of specimens was conducted in an electric oven at 45 °C, after which they were preserved in sealed plastic bags with silica gel (Yu et al. 2020). Voucher specimens are deposited in the Herbarium of the Changbai Mountain Nature Reserve (ANTU) under FCAS accession numbers.

Microscopic observations were performed on hand-cut sections of pileipellis, stipitipellis, lamellae and trama tissues, mounted in 5% KOH and examined with an Olympus CX 23 microscope. Key microstructures, including basidiospores, basidia, cheilocystidia, pleurocystidia, caulocystidia, pileipellis and stipitipellis, were captured photographically and measured. All measurements were taken from 5% KOH-mounted sections, with basidiospore dimensions excluding the apiculus and basidial measurements excluding sterigmata.

Spore data are presented following the format [n/m/p], where n corresponds to the total number of spores measured, m to the number of basidiomata examined, and p to the number of collections (Ge et al. 2021; Liu et al. 2021). Spore size ranges are expressed as (A) c–d (B), where A and B are the minimum and maximum values observed, and c and d denote the 5^th^ and 95^th^ percentiles, respectively. The Q ratio represents the length-to-width ratio of an individual spore, while Qm denotes the average Q value across all spores measured.

### DNA extraction, PCR amplification and sequencing

Total genomic DNA was isolated from dried herbarium specimens with the NuClean Plant Genomic DNA Kit (CWBIO, Beijing, China), adhering to the manufacturer’s protocol. Three nuclear regions, namely the internal transcribed spacer (ITS), the large subunit (LSU), and the protein-coding gene *rpb2*, were selected for amplification.

PCR reactions were conducted in 25 µL reaction mixtures comprised of 12.5 µL of 2× Taq Plus MasterMix (with dye), 1 µL each of forward and reverse primers (10 µM), 1 µL of template DNA, and 9.5 µL of ddH_2_O. Amplification employed the primer pairs ITS1F/ITS4 for ITS (White et al. 1990; Gardes and Bruns 1993), LR0R/LR7 for LSU (Vilgalys and Hester 1990), and bRPB2-6F/bRPB2-7.1R for *rpb2* (Matheny 2005). For ITS and LSU, the thermocycling conditions consisted of an initial denaturation step at 94 °C for 1 min, then 35 cycles of 95 °C for 30 s, 52 °C for 1 min, and 72 °C for 1 min, followed by a final extension at 72 °C for 10 min. The *rpb2* amplification used a similar thermal profile, except that the annealing temperature was lowered to 50 °C and the final extension was reduced to 8 min. All amplicons were visualized on 1% agarose gels and subsequently sent to Sangon Biotech (Shanghai, China) for purification and bidirectional Sanger sequencing.

### Phylogenetic analyses

The final dataset included both newly generated sequences from our collections and publicly available data retrieved from GenBank, with emphasis on previously described taxa of sect. *Marginatae* and affiliated lineages (Horak et al. 2015; Kuo and Matheny 2015; Franchi et al. 2016; Latha and Manimohan 2017; Matheny and Bougher 2017; Larsson et al. 2018; Bandini et al. 2022; Esteve-Raventós et al. 2022; Matheny et al. 2022; Gao et al. 2025). Sequence alignment was carried out using the MAFFT online server (https://mafft.cbrc.jp/alignment/server/; Katoh et al. 2019) with the E-INS-i strategy, followed by manual refinement in BioEdit (Hall 1999). The three gene fragments, ITS, LSU, and *rpb2*, were concatenated using MEGA v5.0 (Tamura et al. 2011). *Nothocybe distincta* (K.P.D. Latha & Manim.) Matheny & K.P.D. Latha served as the outgroup.

Maximum Likelihood (ML) analysis was conducted with IQ-TREE (Trifinopoulos et al. 2016), employing ModelFinder for selecting the optimal substitution model for each partition and 1,000 standard bootstrap replicates (using the -b option). Bayesian Inference (BI) was implemented in MrBayes v3.2.7a (Ronquist et al. 2012), with models determined by MrModeltest v2.3 (Nylander 2004). MCMC analysis was run for a maximum of 10 million generations, sampling trees every 1,000 generations, with automatic termination when the average standard deviation of split frequencies fell below 0.009. The first 25% of sampled trees were discarded as burn-in. Posterior probabilities (PP) were derived from the remaining trees.

## Results

### Molecular phylogeny

A total of 85 samples, including 27 newly obtained sequences from the present study (Suppl. material 1), were analyzed. The final concatenated dataset comprised three gene fragments with a total aligned length of 2920 bp: ITS (830 bp), *rpb2* (740 bp), and LSU (1,350 bp). For ML inference, IQ-TREE was used with ModelFinder selecting TVM+F+I+G4 for *rpb2*, TIM3+F+I+G4 for ITS, and TN+F+I+G4 for LSU; the ML tree had a log-likelihood score of −22,592.5749. For BI, MrBayes was run with the GTR+I+G model selected by MrModeltest for each partition. The MCMC analysis was run for 825,000 generations, sampling trees every 1,000 generations, with a burn-in of 25%. Convergence was confirmed by an average standard deviation of split frequencies of 0.008911, with all ESS values exceeding 200 (minimum = 257) and all PSRF values approaching 1.0 (maximum = 1.005), indicating adequate sampling and convergence.

**Figure 1.**
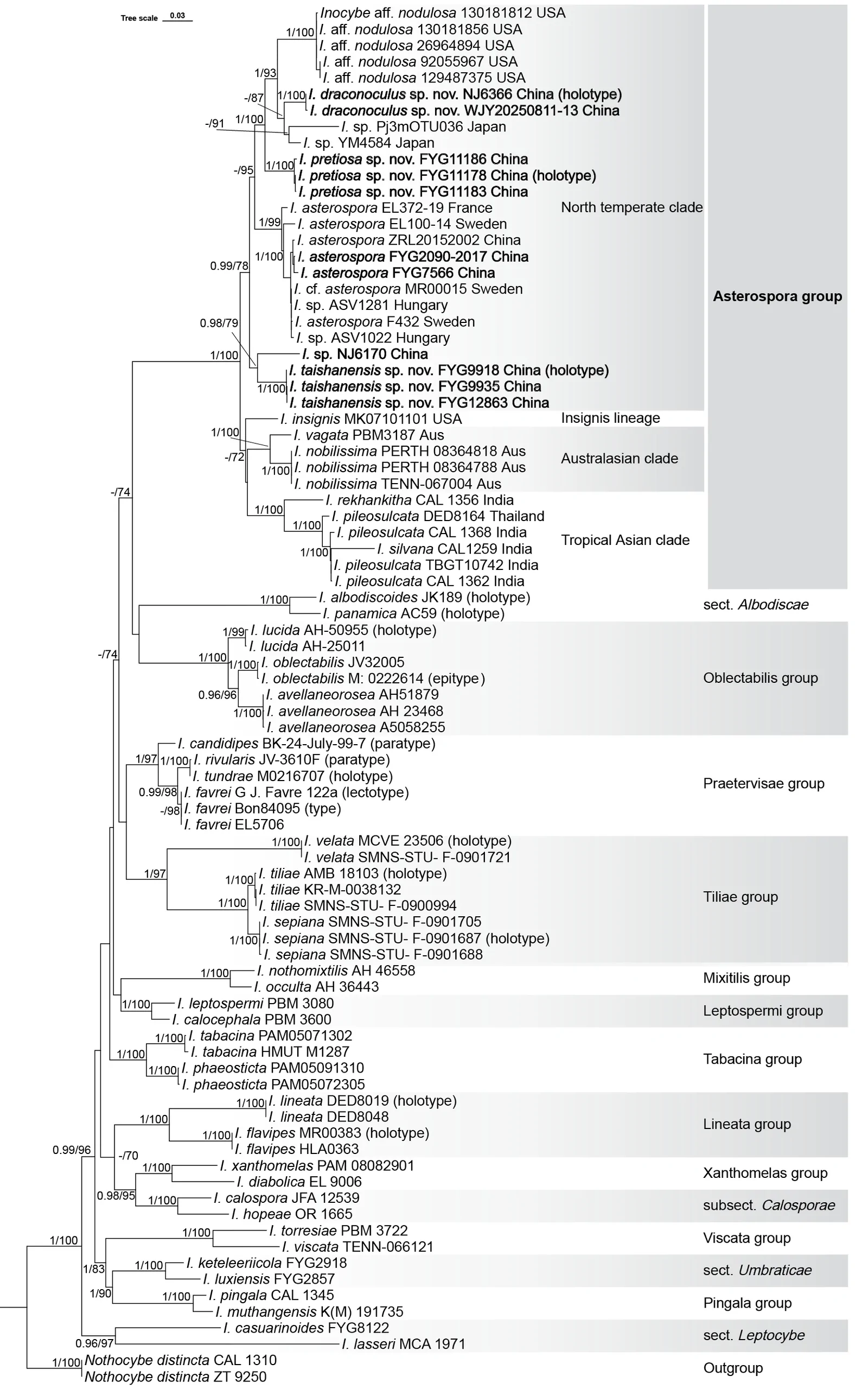
Maximum likelihood phylogeny of the Asterospora group and related lineages based on ITS, LSU and *rpb2* sequences. Bootstrap values (≥ 70%) and Bayesian posterior probabilities (≥ 0.95) are shown near branches. The scale bar indicates substitutions per site. New species are in bold.

Maximum Likelihood (ML) and Bayesian Inference (BI) analyses produced largely congruent topologies; only the ML tree is presented in Fig. 1. In the concatenated phylogeny, the ingroup taxa were resolved into 15 well-supported clades: sect. *Umbraticae* Y.G. Fan, Matheny & T. Bau (PP/BS = 1.00/100), the *Pingala* group (1.00/100), the *Viscata* group (1.00/100), the *Lineata* group (1.00/100), the *Xanthomelas* group (1.00/100), subsect. *Calosporae* (J.E. Lange) Bon (1.00/100), the *Tabacina* group (1.00/100), the *Praetervisae* group (1.00/97), the *Mixilitis* group (1.00/100), the *Leptospermi* group (1.00/100), the *Tiliae* group (1.00/97), the *Oblectabilis* group (1.00/100), sect. *Albodiscae* Matheny (1.00/100), the *Asterospora* group (1.00/100), and sect. *Leptocybe* Takah. Kobay. (1.00/100). Among these, sect. *Leptocybe* is characterized by the absence of caulocystidia on the stipe surface, whereas the remaining 14 clades share nodulose-spored basidiospores and the presence of caulocystidia at least on the upper part of the stipe.

The *Xanthomelas* group and subsect. *Calosporae* formed a strongly supported clade (PP/BS = 0.98/95). Sect. *Albodiscae* and the *Oblectabilis* clade showed a tendency to cluster together, albeit with limited support (PP/BS = 0.53/68). This weakly supported clade, together with the *Asterospora* clade, formed a larger assemblage, a relationship that received support only from ML (PP/BS = 0.94/85). Notably, species in these three clades often share a pinkish to brownish stipe pigmentation, a character that may warrant further morphological and evolutionary investigation.

Within the *Asterospora* group, four distinct subclades can be clearly resolved: the Australasian clade comprising *I. nobilissima* and *I. vagata* (PP/BS = 1.00/100), the tropical Asian clade including *I. pileosulcata*, *I. silvana*, and *I. rekhankitha* (PP/BS = 1.00/100), the Insignis lineage, and the north temperate clade (PP/BS = 0.99/78) encompassing *I. asterospora*, the three new species proposed herein (*I. draconoculus*, *I. pretiosa*, and *I. taishanensis*), together with several undescribed phylogenetic taxa. These four subclades each show a distinct geographic distribution; nevertheless, the phylogenetic relationships among them remain statistically unsupported.

The three new species, *I. draconoculus*, *I. pretiosa*, and *I. taishanensis*, each occupy distinct phylogenetic positions, supporting their novelty from a molecular perspective. Two Chinese collections, FYG7566 and FYG2090-2017, formed a strongly supported clade together with seven samples of *I. asterospora* from both Europe and China. Although some genetic differentiation was observed within this clade, we tentatively interpret this as intraspecific variation, given the concordant morphological features shared by these collections.

### Taxonomy

#### Inocybe asterospora

Taxon classificationFungiAgaricalesInocybaceae

Quél., Bull. Soc. Bot. France 26: 50 (1880)

E29BA3E6-F9DD-523A-9E71-0A690C9AB386

Figs 2, 3

≡ Agaricus asterosporus (Quél.) Cooke, Ill. Brit. Fung. (London) 3: pl. 385 (430) (1884)
≡ *Clypeus asterosporus* (Quél.) P. Karst., Bidr. Känn. Finl. Nat. Folk 48: 213 (1889)
≡ *Astrosporina asterospora* (Quél.) Rea, Brit. basidiomyc. (Cambridge): 210 (1922)

##### Chinese name.

星孢丝盖伞 (Xīng bāo sī gài săn).

##### Description.

***Basidiomata*** medium-sized. Pileus 29–40 mm diam., campanulate or conical when young, then conico-campanulate or umbrella-shaped, with a distinct umbo at center when mature; margin slightly incurved to expanding. Surface dry, silky-smooth when young, sometimes moist, cutis tightly attached to pileus, fibrillose, then radially finely rimulose, becoming radially streaked and cracked at margin when mature; dark brown (6D6) to brownish-yellow (6E6) at center, margin yellowish-brown (6E6). ***Lamellae*** 4 mm wide, adnexed, rather crowded, unequal in length, with 3–4 tiers of lamellulae, edge irregularly serrulate, with vertical striae near pileus side and rounded spots near edge; white (1A1) when young, then grayish-white (5A1) to pale yellowish-brown (5B3), vertical striae white (1A1), rounded spots pale brownish (5C6). ***Stipe*** 62–72 mm long, 4–5 mm wide, cylindrical, with longitudinal striae downwards, solid, base subclavate-swollen, marginately bulbous, up to 10–12 mm wide, with a volva-like structure formed by white mycelium at the margin, surface covered with white pruina; yellowish-brown (6C3) to dark brown (6D6). ***Context*** fleshy in pileus, white (1A1); in stipe fibrillose, pale brownish (5B4) to pale brown (6C6) near surface, yellowish-white (3A2) to brownish-pink (5A2) internally, white (1A1) at base. ***Odor*** strong spermatic.

**Figure 2.**
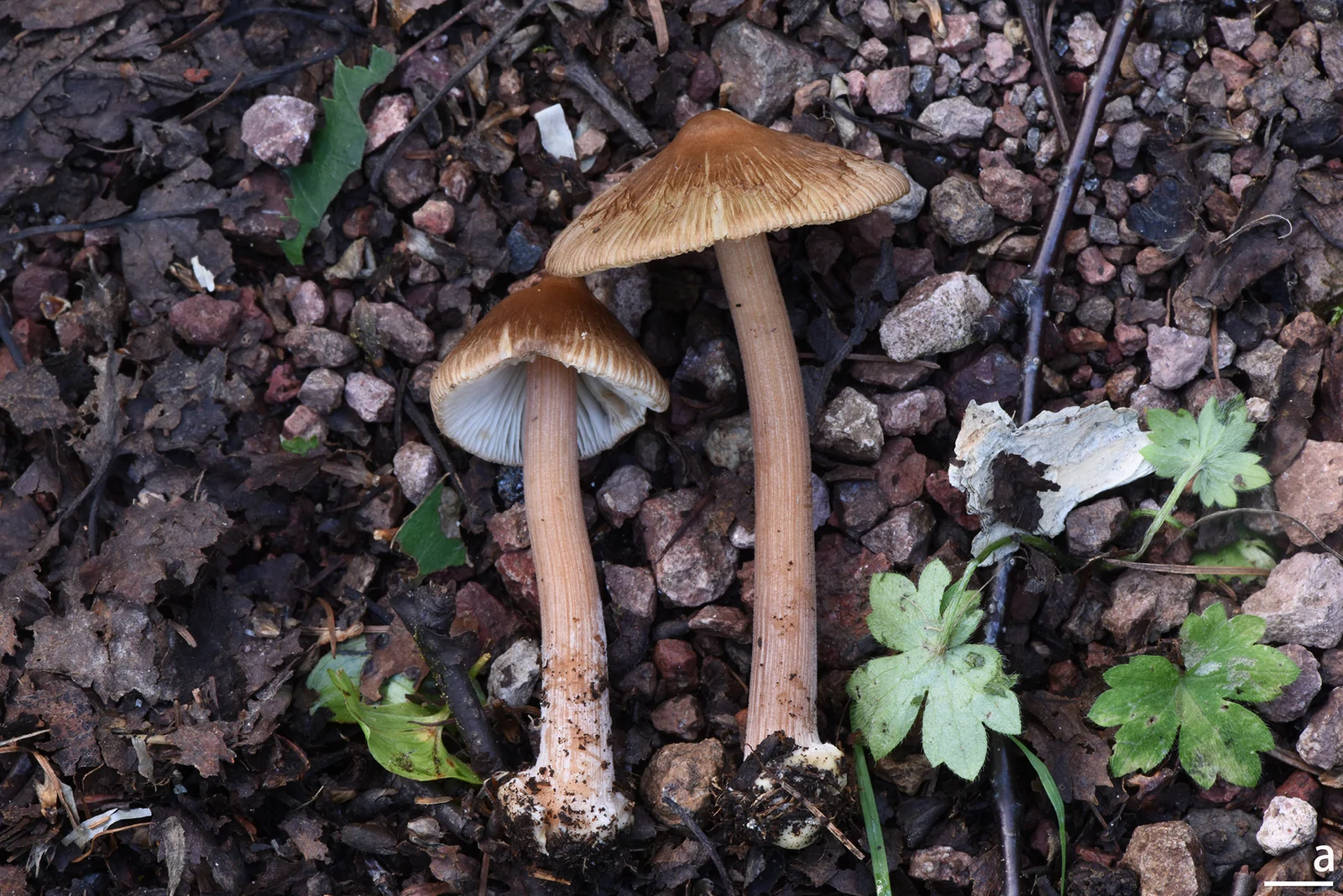
Macroscopic features of *Inocybe asterospora* (FYG2090). **a, b**. Basidiomata *in situ*. Scale bar: 10 mm. Photos by Y.G. Fan.

***Basidiospores*** [100/2/2] (8.2) 9.9–10.87–12.0 (12.8) × (7.6) 8.0–9.10–10.0 (11.0) μm, Q = (1.00) 1.01–1.20–1.40 (1.48), Qm ± SD = 1.20 ± 0.1212, stellate, angular, smooth, with 10–13 angular projections, yellow to ochraceous-yellow in 5% KOH. Basidia 25–39 × 10–19 μm, clavate to broadly clavate, with 2–4 sterigmata 3.5–8 μm long, occasionally 2-spored basidia up to 62 μm long, thin-walled, mostly colorless to pale yellow, usually containing pale yellow or yellowish-green oily-like inclusions, occasionally transparent. Lamellae edge sterile; ***Pleurocystidia*** 60–71–82 × 19–22–31 μm (n = 30), fusiform, broadly fusiform, subutriform, apex slightly acute or obtuse, occasionally encrusted with crystals, base tapering, obtuse or subtruncate, occasionally forming a short pedicel, thick-walled, walls gradually thickening from base to apex, thickest at apex up to 5 μm, walls pale yellow or colorless and transparent. ***Cheilocystidia*** 46–59–70 × 11–20–28 μm (n = 30), mostly similar to pleurocystidia, broadly fusiform, walls pale yellow or colorless. Paracystidia 16–32 × 8–17.5 μm, abundant, pyriform or broadly clavate, clustered, thin-walled, colorless, transparent, without distinctive contents. ***Caulocystidia*** 49–80 × 15–24 μm, extending from upper stipe to base, more abundant on upper part, clustered or solitary, subfusiform or utriform, apex obtuse or subtruncate, smooth, often encrusted with crystals, mostly with walls gradually thickening from base to apex, colourless or pale yellow. Cauloparacystidia 20–25 × 11–15 μm, abundant, subclavate or ellipsoid, thin-walled, colourless, transparent. ***Hymenophoral trama*** 55–140 μm wide, subregular, composed of broadly cylindrical hyphae 11–25 μm wide, walls slightly thickened, 1.5 μm, smooth, colourless, some slightly rough. ***Pileipellis*** a cutis, 52–130 μm thick, yellowish-brown to yellow-brown, composed of elongate cylindrical hyphae, occasionally uplifted, 3.5–11.5 μm wide, thin-walled, surface rough, pale yellow. ***Pileal trama*** subregular, composed of inflated hyphae 10–25 μm diam., smooth, thin-walled, transparent, colorless. ***Stipitipellis*** regularly arranged, rather smooth, hyphae narrowly elongate, 2–8 μm wide, mostly smooth, pale yellow to yellow. ***Stipe trama*** colorless, elongate-cylindrical, regularly arranged, 7–18 μm wide, thin-walled, smooth, colorless. ***Oleiferous hyphae*** 3–11 μm wide, common in hymenium, pileus and stipe, sometimes branched, with tuberculate nodules, smooth, pale yellow to bright yellow. ***Clamp connections*** present in all tissues.

**Figure 3.**
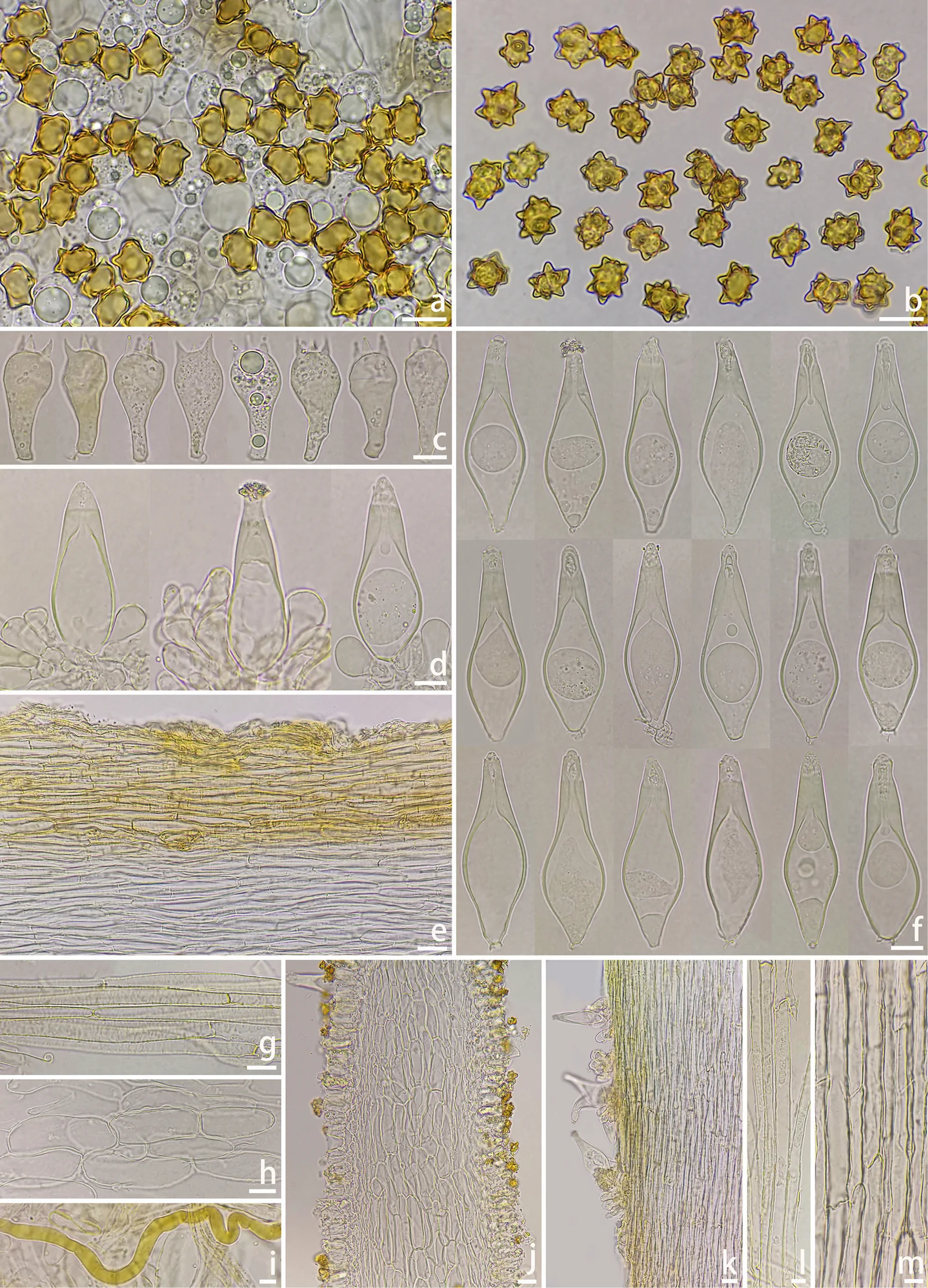
Microscopic morphology of *Inocybe asterospora*. **a, b**. Spores; **c**. Basidia; **d**. Cheilocystidia; **e**. Pileipellis; **f**. Pleurocystidia; **g**. Pileipellis hyphae; **h**. Pileal trama hyphae; **i**. Oleiferous hyphae; **j**. Hymenophoral trama; **k**. Caulocystidia on stipe; **l**. Stipitipellis hyphae; **m**. Stipe trama hyphae. Scale bars: 10 µm (**a–d, f–h, m**); 20 µm (**e, j, k**); 5 µm (**i, l**). Photographed by Ren Wanyi.

##### Habitat.

Under *Corylus*, *Quercus* spp., and *Betula ermarnii* in East Asia, associated with *Corylus*, *Quercus* spp., *Fagus sylvatica*, and *Carya* spp. in Europe.

##### Geographical distribution.

East Asia (Kobayashi 2002; Bau 2022), Europe (Quélet 1879; Jacobsson 2008), North America (Kauffman 1918). Currently known from Beijing, Inner Mongolia, Jilin, Ningxia, and Shaanxi in China.

##### Specimens examined.

China • Jilin Province: Yanbian Korean Autonomous Prefecture, Antu County, Erdaobaihe Town, northern slope of Changbai Mountain, 42°02'57"N, 128°04'01"E, al. 740 m, under *Quercus* trees, 1 September 2022, leg. Y.G. Fan and W.J. Yu, FYG7566 (FCAS4246); • 42°03'00"N, 128°03'36"E, al. 1962 m, in *Betula ermarnii* forests, 28 Aug 2017, Y.G. Fan, FYG2090 (FCAS4245); • Dunhua City, Tuanbei forest farm, 44°01'41"N, 127°55'01"E, al. 766 m, in *Quercus mongolica* forests, 16 Aug 2016, Y.G. Fan, FYG2016060 (FCAS4473). • Ningxia Hui Autonomous Region: Guyuan City, Jingyuan County, Jingheyuan Town, Liupanshan National Forest Park, 35°25'27"N, 106°24'37"E, al. 1824 m, in forest of *Quercus liaotungensis* Koidz.*‌*, 12 Aug. 2025, Y.G. Fan & W.J. Yu, FYG12384 (FCAS4474).

##### Remarks.

*Inocybe asterospora* is distinguished by its medium-sized basidiomata, a brown pileus that is prominently rimulose to rimose, a flesh-brown to pinkish stipe with a distinctly marginate bulbous base, and a strongly spermatic odor. Ecologically, it is closely associated with members of *Fagaceae* and *Betulaceae*. The species is widely distributed across the north temperate zone (Kauffman 1918; Jacobsson 2008; Bau 2022), although sporadic records from tropical and Southern Hemisphere regions require further confirmation owing to the lack of molecular evidence. In China, *I. asterospora* has been reported from numerous provinces spanning temperate to subtropical zones (Fan and Bau 2010); however, the distribution confirmed by both morphological examination and molecular data based on voucher specimens is currently confined to northern China. A fatal poisoning case was documented in Zhuzhou County, Hunan Province, in July 1985, in which three of five individuals died after consuming wild mushrooms identified as *I. asterospora*; symptoms included headache, nausea, vomiting, diarrhoea, salivation, profuse sweating, chest tightness, convulsions, haematemesis, bloody stools, dyspnoea and coma (Chen et al. 1987). Notably, our recent chemical analysis detected both muscarine and psilocybin in *I. asterospora*, representing the first report of psilocybin in the nodulose-spored lineage of *Inocybe* (Liang et al. 2026).

#### Inocybe taishanensis

Taxon classificationFungiAgaricalesInocybaceae

Y.G. Fan, Y.P. Ge & W.Y. Ren sp. nov.

731769A1-F47C-5E53-82DC-A329B71A2ABD

864902

Figs 4, 5

##### Chinese name.

泰山丝盖伞 (Tài Shān sī gài săn).

##### Etymology.

*taishanensis* refers to Mount Tai (Tài Shān) Scenic Area, Tai’an City, Shandong Province, China, the type locality of this species.

##### Diagnosis.

Basidiomata medium-sized, pileus umbonate, margin distinctly uplifted when old, surface rimulose with yellowish-brown radical streaks; basidiospores 8–13.5 × 6.5–12.6 μm, oblong with distinct angular nodules, apical nodules distinctly elongated; hymenial cystidia utriform, walls pale yellow. Macroscopically most similar to *I. asterospora*, but differs in having oblong basidiospores and lageniform hymenial cystidia with a more distinct neck and pale yellow walls.

##### Holotype.

China • Shandong Province: Tai’an City, Mount Tai Scenic Area, 36°15'25"N, 117°06'12"E, al. 462 m, in broad-leaved forests dominated by *Quercus acutissima* Carruth., 20 August 2023, leg. Y.P. Ge, FYG9918 (FCAS4124).

##### Description.

***Basidiomata*** medium-sized. ***Pileus*** 32–45 mm diam., campanulate or conical when young, then conico-campanulate or applanate, with a distinct umbo at center when mature; margin slightly decurved when young, uplifted when old; surface dry, silky-smooth when young, sometimes moist, fibrillose, then coarsely rimulose, becoming radially rimose towards the margin with age, sometimes distinctly cracked; center darker, grayish-brown (6C4); margin pale yellowish-brown (5B5). ***Lamellae*** 3–4 mm wide, adnexed, moderately crowded, unequal in length, with 2–3 tiers of lamellulae, edge uneven, irregularly serrulate; white (1A1) when young, then dirty white (1A2) to pale yellowish-brown (5B4), yellowish-brown (5D6) to dark brown (5C7) upon drying. ***Stipe*** 38–56 mm long, 3–4 mm wide, cylindrical, solid, base with a marginate bulb up to 7 mm wide; surface entirely pruinose and forming longitudinal striae; yellowish-brown (4A3) to brown (5C8). ***Context*** fleshy in pileus, white (1A1); in stipe fibrillose, pale brown (5B4) to pale brownish (6C6) near surface, yellowish-white (3A2) to white (1A1) internally, white (1A1) at base, firm. ***Odor*** spermatic.

**Figure 4.**
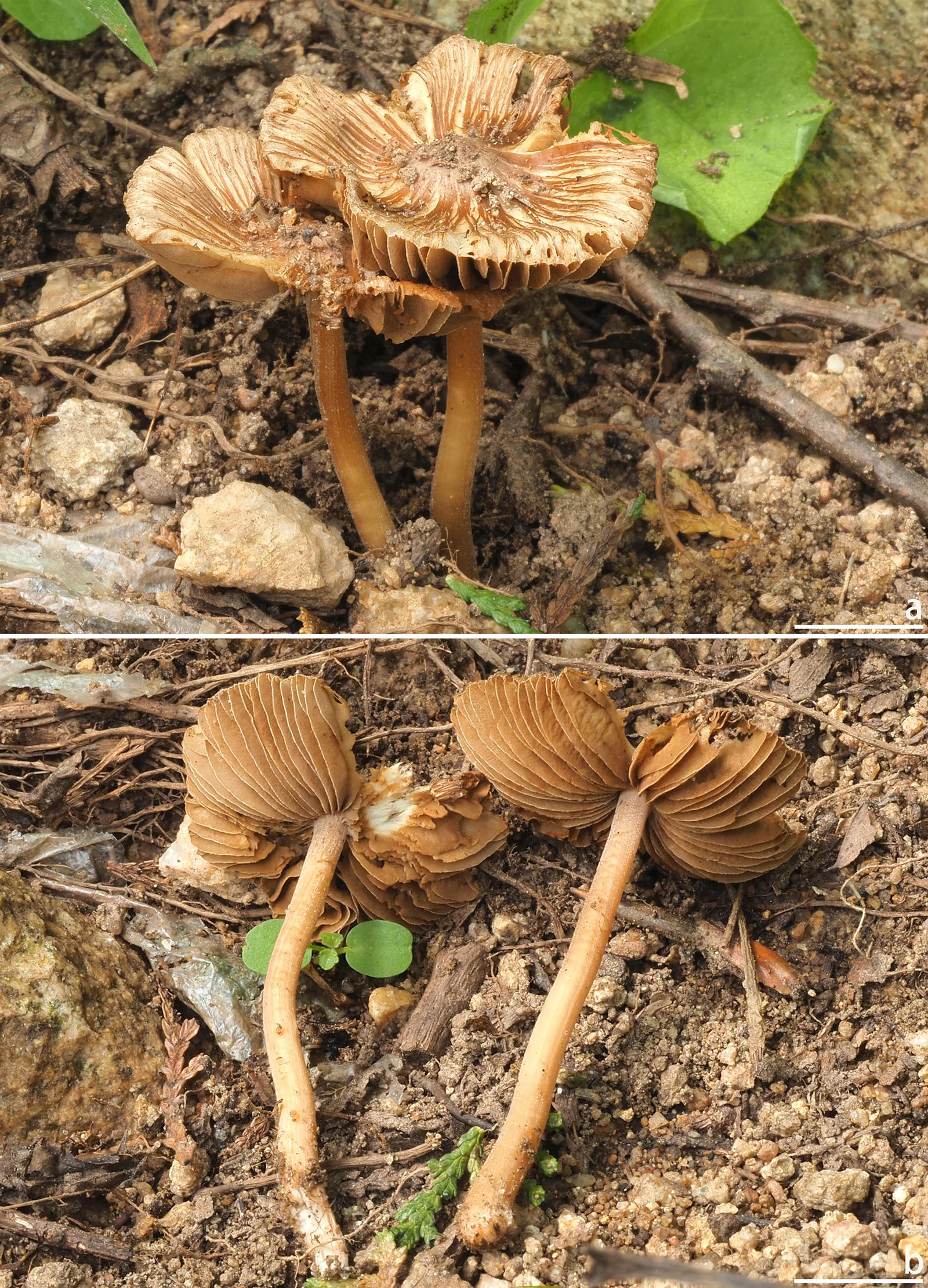
Macroscopic features of *Inocybe taishanensis* (FYG9918, holotype). **a, b**. Basidiomata *in situ*. Scale bars: 15 mm (**a, b**). Photos by Y.P. Ge.

***Basidiospores*** [100/3/3] (8.0) 9.8–10.9–12.5 (13.5) × (6.5) 6.8–8.1–9.8 (12.6) μm, Q = (1.00) 1.11–1.35–1.69 (1.76), Qm ± SD = 1.35 ± 0.166, angular to rectangular-angular, with 10–13 angular nodules, some spores with apical nodules distinctly elongated, yellow to ochraceous-yellow in 5% KOH. Basidia 21–37 × 10–15 μm, narrowly clavate to broadly clavate, with 2–4 sterigmata 3–8 μm long, thin-walled, mostly subhyaline to pale yellow, usually containing small vesicular pale yellow or yellow inclusions, occasionally transparent. ***Pleurocystidia*** 52–70–85 × 16–23–28 μm (n = 30), fusiform, broadly fusiform, subutriform, colorless to pale yellow, occasionally with colorless to pale yellow small vesicles, apex obtuse, occasionally encrusted with crystals, sometimes bent to one side, base tapering, obtuse or subtruncate, occasionally forming a short pedicel; thick-walled, thickest at apex up to 3 μm, walls pale yellow. ***Cheilocystidia*** 50–64–79 × 16–22–33 μm (n = 30), mostly similar to pleurocystidia, narrowly fusiform to broadly fusiform, walls pale yellow or subhyaline. ***Cheiloparacystidia*** 11–27 × 9–14 μm, abundant, pyriform or broadly clavate, clustered, apex obtuse, thin-walled, colorless, transparent, without distinctive contents. ***Caulocystidia*** 32–60 × 15–23 μm, descending to stipe base, mostly present on upper stipe, sparser downwards, solitary or clustered, subfusiform, apex obtuse or subtruncate, smooth, often encrusted with crystals, walls subhyaline or pale yellow. ***Cauloparacystidia*** 21–38 × 12–20 μm, abundant, subellipsoid or short-clavate, apex obtuse, thin-walled, colorless, transparent. ***Hymenophoral trama*** 56–230 μm wide, subregular, composed of broadly cylindrical to inflated hyphae 8–35 μm wide, walls slightly thickened, smooth, colorless, some slightly rough. ***Pileipellis*** a cutis, 40–115 μm thick, yellowish-brown to yellow-brown, composed of elongate cylindrical hyphae, occasionally uplifted, 3–14 μm wide, thin-walled, finely encrusted, pale yellow. ***Pileal trama*** subregular, composed of cylindrical hyphae 11–25 μm wide and inflated hyphae 31–41 μm wide, smooth, thin-walled, transparent, colorless. ***Stipitipellis*** regularly arranged, rather smooth, hyphae narrowly cylindrical, 3–7 μm wide, mostly smooth, pale yellow to yellow. ***Stipe trama*** colorless, hyphae elongate-cylindrical, regularly arranged, 10–16 μm wide, thin-walled, smooth, colorless. ***Oleiferous hyphae*** common in hymenophoral trama, pileus and stipe, 2–13 μm wide, sometimes branched, with tuberculate nodules, smooth, pale yellow to bright yellow. ***Clamp connections*** present in all tissues.

**Figure 5.**
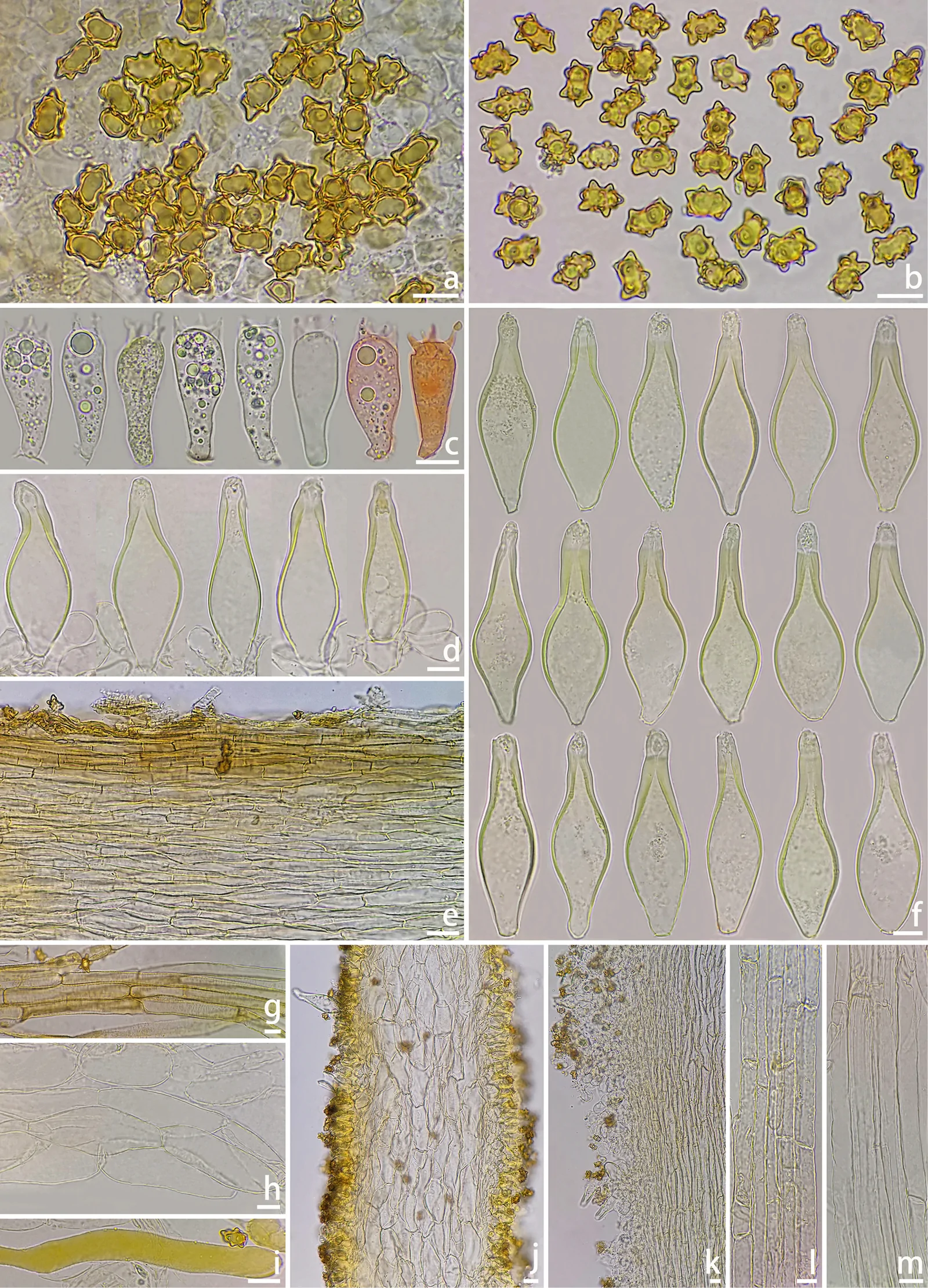
Microscopic features of *Inocybe taishanensis* (FYG9918, holotype). **a, b**. Basidiospores; **c**. Basidia; **d**. Cheilocystidia and cheiloparacystidia; **e**. Pileipellis and part of the pileal trama; **f**. Pleurocystidia; **g**. Pileipellis hyphae; **h**. Pileal trama hyphae; **i**. Oleiferous hyphae; **j**. Cross section of lamellae; **k**. Caulocystidia on stipe surface; **l**. Stipitipellis hyphae; **m**. Stipe trama hyphae. Scale bars: 10 μm (**a–d, f, g, k**); 20 μm (**e, h, i**); 5 μm (**l, m**). Photos by W.Y. Ren.

##### Habitat.

Solitary or scattered in broad-leaved forests dominated by *Quercus acutissima*.

##### Geographical distribution.

Known only from Shandong Province, China.

##### Additional specimens examined.

China • Shandong Province: Tai’an City, Mount Tai Scenic Area, 36°15'25"N, 117°06'12"E, al. 460 m, in broad-leaved forests dominated by *Quercus acutissima*, 2 September 2023, leg. Y.P. Ge & Y.G. Fan, FYG9935 (FCAS4470); • Zulaishan National Forest Park, 36°02'24"N, 117°13'50"E, al. 521 m, 11 September 2025, Y.P. Ge & Y.G. Fan, FYG12863 (FCAS4471).

##### Remarks.

This species is currently known only from Tai’an City, Shandong Province. It is characterized by a broad pileus with a rough, cracked surface, angular to rectangular basidiospores, and hymenial cystidia with pale yellow walls. Compared with *I. asterospora*, both have a radially streaked pileus with a finely rimulose to cracked surface and an entirely pruinose stipe. However, *I. asterospora* has a stouter stipe that is brownish or pinkish-brown, typically stellate basidiospores, and longer hymenial cystidia with colorless walls (Stangl 1989; Kobayashi 2002; Jacobsson 2008). In our three-gene phylogenetic analysis, *I. taishanensis* forms a sister lineage to an independent clade represented by a single specimen (NJ6107) collected from *Quercus* forests in Hubei Province. Unfortunately, NJ6107 is immature, and additional collections are needed to complete its morphological characterization.

#### Inocybe draconoculus

Taxon classificationFungiAgaricalesInocybaceae

Y.G. Fan & Y.P. Ge sp. nov.

AA1DB768-C574-551E-949C-B627B77D2541

864903

Figs 6, 7

##### Chinese name.

龙睛丝盖伞 (Lóng Jīng sī gài săn).

##### Etymology.

The specific epithet *draconoculus* is used as a noun in apposition, derived from Latin *draco* (dragon) and *oculus* (eye), referring to the stellate, nodulose basidiospores, which resemble a dragon’s eye. The name also alludes to the cultural imagery of Wufeng County, Hubei Province, where the type specimen was collected.

##### Diagnosis.

Basidiomata medium-sized, pileus umbonate, margin slightly incurved, surface with pale brownish radial streaks and fine, even rimulose cracks; basidiospores 9.6–12.0 × 8.2–10.2 μm, stellate, with conspicuous angular nodules; hymenial cystidia utriform, thick-walled, walls hyaline. Most similar to *I. asterospora*, but differs by the smaller basidiomata, darker brown pileus, more slender stipe, hymenial cystidia with a more distinct neck, and occurrence in subtropical evergreen broad-leaved forests.

**Figure 6.**
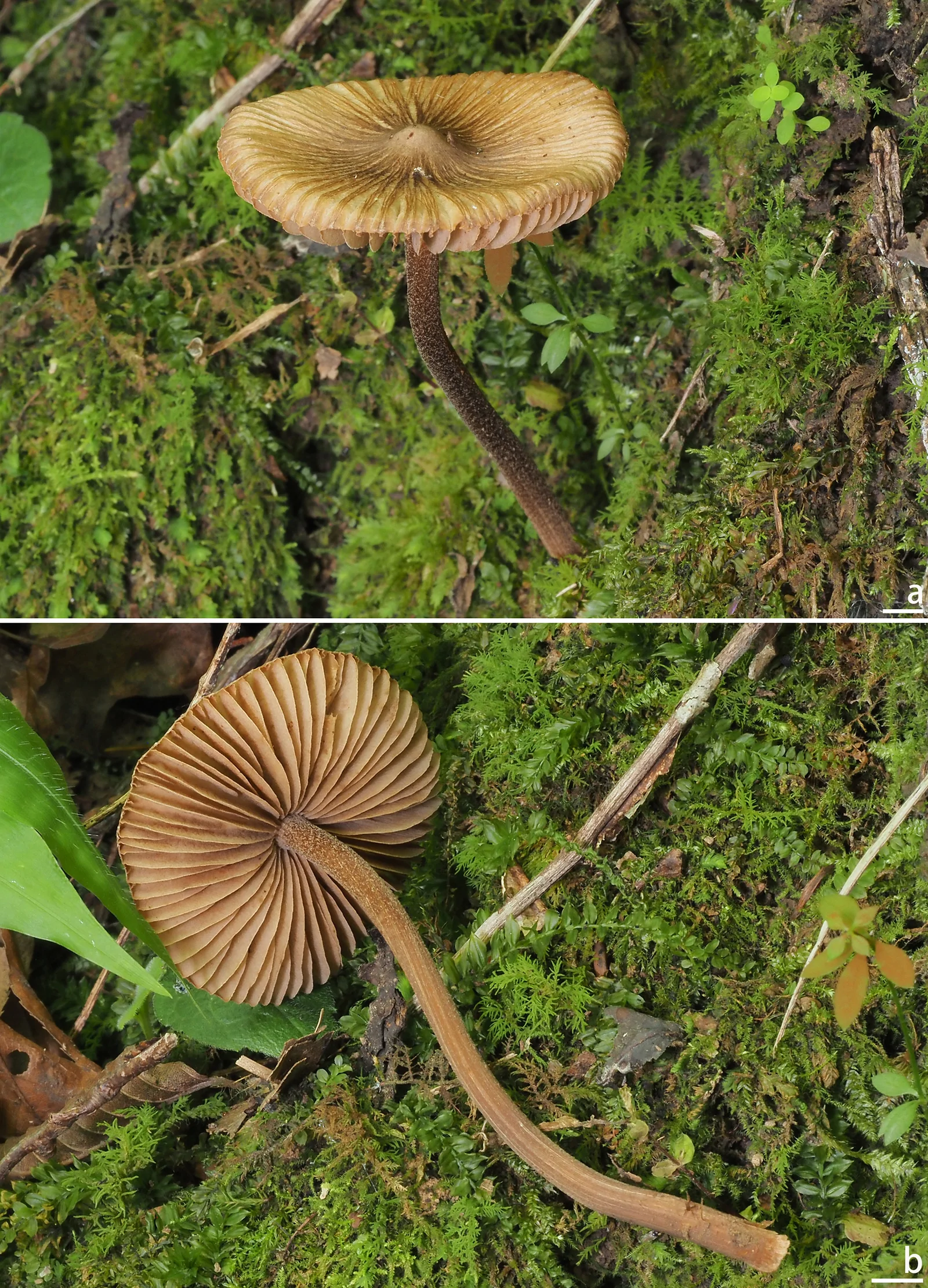
Macroscopic features of *Inocybe draconoculus* (FCAS4253, holotype). **a, b**. Basidiomata *in situ*. Scale bars: 5 mm (**a, b**). Photos by Y.P. Ge.

##### Holotype.

China • Hubei Province: Yichang City, Wufeng Tujia Autonomous County, 30°09'52"N, 110°41'06"E, al. 1105 m, under *Quercus* forests, 9 August 2024, leg. Y.P. Ge, Q. Na, T. Wang, J.W. Guo & Y.L. Gai, NJ6366 (FCAS4253).

##### Description.

***Basidiomata*** medium-sized. Pileus 24–39 mm diam., campanulate or conical when young, then plano-concave, with a distinct small umbo at center when mature, the entire pileus uplifted so that the area around the umbo is depressed, margin slightly incurved; surface dry, silky-smooth when young, fibrillose, then becoming evenly radially finely rimulose, margin cracked when old; center grayish-brown (2B3) to pale brownish (5C4), margin pale brown (6C3). ***Lamellae*** 3–4 mm wide, adnexed, rather crowded, unequal in length, with 2–3 tiers of lamellulae, edge irregularly serrulate; white (1A1) when young, then pale brown (6D5) to yellowish-brown (5B3). ***Stipe*** 42–65 mm long, 3–4 mm wide, cylindrical, equal, solid, base marginately bulbous; surface covered with white pruina, longitudinally striate; color uniform, yellowish-brown (5B4) to brown (5D6), longitudinal striae yellowish-white (1A2). ***Context*** fleshy in pileus, white (1A1); in stipe fibrillose, pale brownish (5B4) to pale brown (6C6) near surface, dirty white (3A2) to white (1A1) internally, white (1A1) at base. ***Odor*** spermatic.

**Figure 7.**
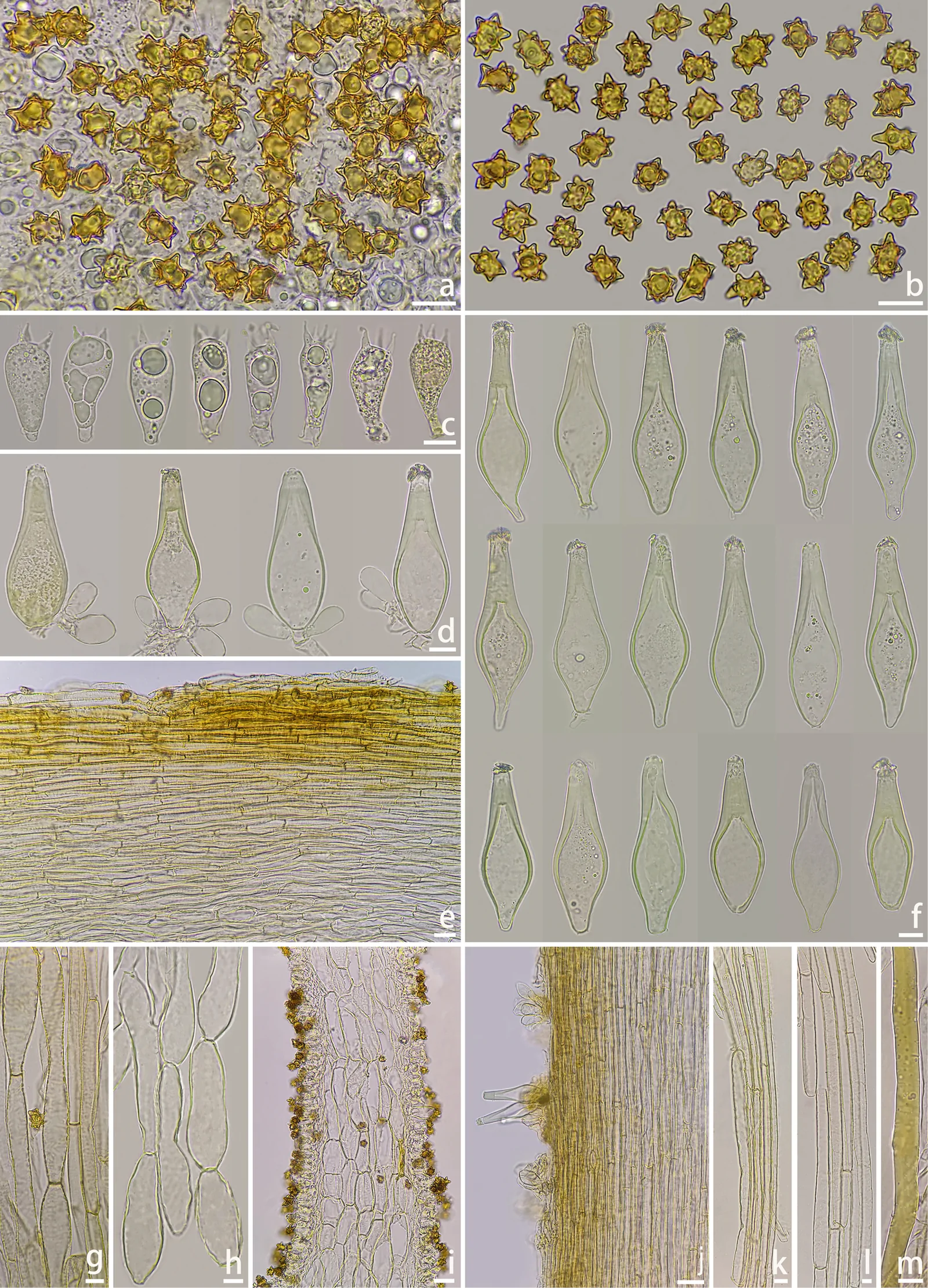
Microscopic features of *Inocybe draconoculus* (FCAS4253, holotype). **a, b**. Basidiospores; **c**. Basidia; **d**. Cheilocystidia and cheiloparacystidia; **e**. Pileipellis and part of the pileal trama; **f**. Pleurocystidia; **g**. Pileipellis hyphae; **h**. Pileal trama hyphae; **i**. Cross section of lamellae; **j**. Caulocystidia on stipe surface; **k**. Stipitipellis hyphae; **l**. Stipe trama hyphae; **m**. Oleiferous hyphae. Scale bars: 10 μm (**a–d, f, g, k**); 20 μm (**e, h–j**); 5 μm (**l, m**). Photos by W.Y. Ren.

***Basidiospores*** [100/2/2] (9) 9.6–10.75–12.0 (15) × (8) 8.2–9.52–10.2 (13) μm, Q = (0.85) 1.00–1.13–1.35 (1.41), Qm ± SD = 1.13 ± 0.108, stellate, smooth, with 11–13 angular projections, yellow to bright yellow in 5% KOH, occasionally one spore with a slightly elongated nodule. Basidia 21–43 × 8–15 μm, clavate to broadly clavate, some gradually tapering downwards, with 2–4 sterigmata 2–8 μm long, thin-walled, mostly subhyaline to yellowish-green, usually containing pale yellow or yellowish-green oily-like inclusions, occasionally transparent. Lamellae edge sterile; ***Pleurocystidia*** 64–76–88 × 16–22–28 μm (n = 30), sublageniform to lageniform, occasionally fusiform to broadly fusiform, apex obtuse or subtruncate, often encrusted with crystals, base tapering, mostly forming a short pedicel, sometimes obtuse or subtruncate, thick-walled, thickest at apex up to 4 μm, walls mostly subhyaline. ***Cheilocystidia*** 55–67–78 × 18–22–27 μm (n = 30), similar to pleurocystidia, broadly fusiform or utriform, base mostly obtuse, walls pale yellowish-green. Paracystidia 11–24 × 8–14 μm, abundant, ellipsoid or broadly clavate, clustered, thin-walled, colorless, transparent, without distinctive contents. ***Caulocystidia*** 45–73 × 13–25 μm, extending from the upper stipe to the base, more abundant on the upper part, clustered or solitary, subfusiform or utriform, apex obtuse or subtruncate, smooth, often encrusted with crystals, walls colorless. Cauloparacystidia 15–27 × 10–18 μm, abundant, subclavate or ellipsoid, thin-walled, pale yellow, interior colorless, transparent. ***Hymenophoral trama*** 50–135 μm wide, subregular, composed of broadly cylindrical hyphae 10–28 μm wide, thin-walled, smooth, pale yellowish-green, some slightly rough. ***Pileipellis*** a cutis, 50–120 μm thick, bright yellow to yellowish-brown, composed of elongate cylindrical hyphae, occasionally uplifted, 5.5–21 μm wide, thin-walled, rough, pale yellow. ***Pileal trama*** subregular, composed of broadly cylindrical and inflated hyphae 9–35 μm diam., smooth, thin-walled, pale yellowish-green or transparent, colorless. ***Stipitipellis*** regularly arranged, rather smooth, hyphae cylindrical, 4–10 μm wide, thin-walled, mostly smooth, pale yellow to yellow. ***Stipe trama*** colorless, elongate-cylindrical, regularly arranged, 8–21 μm wide, thin-walled, colorless, transparent. ***Oleiferous hyphae*** 3–12 μm wide, common in hymenophoral trama, pileus and stipe, sometimes branched, with tuberculate nodules, smooth, pale yellow to bright yellow. ***Clamp connections*** present in all tissues.

##### Habitat.

Solitary or scattered under *Fagaceae* forests.

##### Geographical distribution.

Known only from Hubei Province, China.

##### Additional specimens examined.

China • Hubei Province: Yichang City, Wufeng Tujia Autonomous County, 30°09'52"N, 110°41'05"E, al. 1109 m, in *Quercus* forests, 11 August 2025, leg. W.J. Yu, WJY20250811-13 (FCAS4472).

##### Remarks.

This species is characterized by a pileus with even, finely rimulose streaks, a plano-concave shape with a distinct acute umbo at the center, stellate basidiospores, and utriform hymenial cystidia. Morphologically, it resembles *I. pileosulcata* in pileus streaking and spore dimensions. However, *I. pileosulcata* has a more conspicuously sulcate and splitting (plicate) pileus surface from the margin to the smooth umbo, paler pileus and stipe colors, smaller spore nodules, mostly fusiform and slightly smaller hymenial cystidia (55–70 × 18–26 μm), and occurs in tropical lowland and montane forests (750–1700 m elev.) dominated by *Dipterocarpus*, *Castanopsis*, *Shorea*, intermixed with *Pinus kesiya* (Horak et al. 2015). Phylogenetically, *I. draconoculus* forms a close alliance with *I. pretiosa*, a species with which it shares a brown to dark brownish pigmentation and a relatively slender basidiome. However, *I. pretiosa* is readily distinguished by its persistently dome-shaped pileus that never becomes fully applanate, a stipe that is typically paler towards the apex, basidiospores with fewer but more prominent and broader nodules, and distinctly larger pleurocystidia.

#### Inocybe pretiosa

Taxon classificationFungiAgaricalesInocybaceae

W.J. Yu & W.Y. Ren sp. nov.

A40B2921-3DF7-5A4A-869A-C488B08E1456

864904

Figs 8, 9

##### Etymology.

*pretiosa* refers to the beautiful and luxurious appearance of this species.

##### Chinese name.

华贵丝盖伞 (Huá guì sī gài săn).

##### Diagnosis.

Basidiomata small; pileus umbonate, surface with dark brown appressed fibrils; stipe surface covered with white pruinose powder, marginately bulbous at base; basidiospores 9.2–13.5 × 7.5–11.5 μm, with conspicuous angular nodules; hymenial cystidia lageniform. Most similar to *I. draconoculus*, but differs by the distinctly smaller basidiomata, darker pileus, fewer spore nodules, and markedly larger hymenial cystidia.

##### Holotype.

China • Shaanxi Province: Baoji City, Mei County, Tangyu Town, Tangyukou, Taibai Mountain National Forest Park, 34°06'10"N, 107°54'06"E, al. 1347 m, under broad-leaved forests of *Corylus chinensis*, *Carpinus cordata*, *Quercus wutaishanica*, 19 August 2024, leg. Y.G. Fan & J.R. Wang, FYG11178 (FCAS4247).

##### Description.

***Basidiomata*** small. Pileus 14–27 mm diam., conical or campanulate when young, then conico-campanulate to convex, with a distinct umbo at center when mature; margin slightly incurved when young, then expanding. Surface dry, silky-smooth when young, then radially rimulose, with radial fine cracks extending from center to margin when mature, cutis easily separable from pileus at maturity; dark brown (3E7) to reddish-brown (5E6) at center, gradually paler outwards, pale brownish (3D6), margin pale yellowish-brown (3A4) to yellowish-brown (3C8). ***Lamellae*** 2–3 mm wide, adnexed, moderately crowded, unequal in length, with 2–3 tiers of lamellulae, edge irregularly serrulate; white (1A1) to pale yellow (1A4) when young, then yellowish-brown (4B5) to brown (4D7) at maturity. ***Stipe*** 44–53 mm long, 1.5–3.0 mm wide, cylindrical, solid, base marginately bulbous, up to 6 mm wide, with a volva-like structure formed by white mycelium at the margin; surface densely covered with white pruina, more abundant on upper part, gradually sparser toward base; yellowish-brown (4B8) to dark brown (5D8), paler in upper-middle part, pale yellowish-brown (2B5) to yellowish-white (3A1). ***Context*** fleshy in pileus, brownish-pink (5A2), brown (5D6) at center, blackish-brown (2F6) at umbo; in stipe fibrillose, brown (6E5) near surface, white (1A1) to grayish-white (1B2) internally and at base. ***Odor*** spermatic.

**Figure 8.**
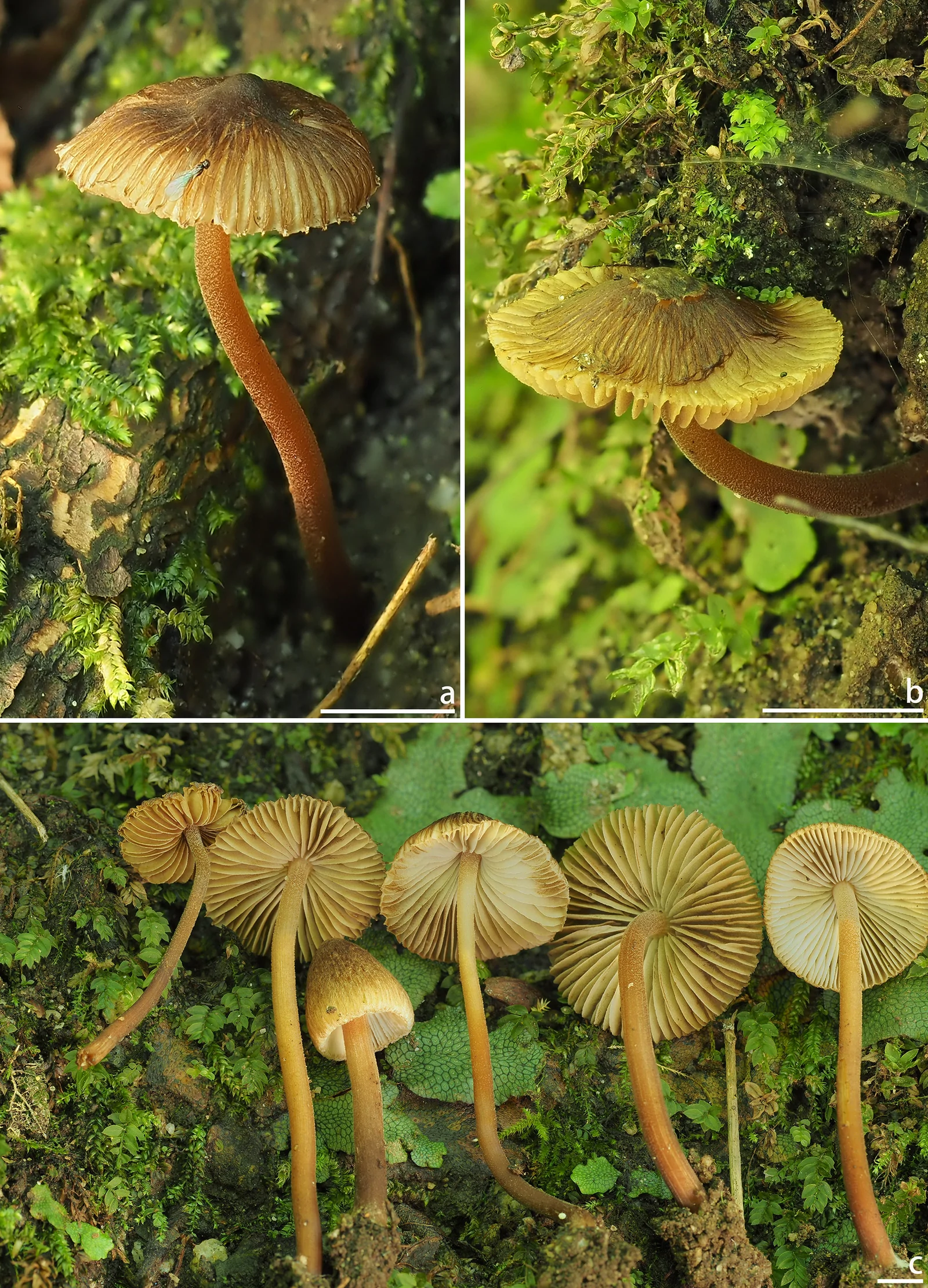
Macroscopic features of *Inocybe pretiosa* (FCAS4247, holotype). **a–c**. Basidiomata *in situ*. Scale bars: 20 mm (**a, b**); 5 mm (**c**). Photos by Y.G. Fan.

***Basidiospores*** [100/3/3] (9.2) 9.7–10.75–12.2 (13.5) × (7.5) 8.5–9.63–10.8 (11.5) μm, Q = (1.00) 1.01–1.12–1.31 (1.36), Qm ± SD = 1.12 ± 0.096, stellate, angular, with 9–10 nodules, ochraceous-yellow to yellowish-brown in 5% KOH. Basidia 23–34 × 11–17 μm, a few up to 59 × 15 μm, clavate to broadly clavate, with 4 or 2 sterigmata 3–7 μm long, sterigmata of 2-spored basidia up to 11 μm long, thin-walled, mostly colorless, greyish or pale yellow, usually containing yellow or yellowish-green inclusions, occasionally transparent. Lamellae edge sterile; ***Pleurocystidia*** 51–80–106 × 20–24–31 μm (n = 30), elongate-fusiform or lageniform with a narrow neck, apex mostly obtuse, often encrusted with crystals, base tapering to subtruncate, occasionally with a short pedicel, walls 2–3 μm thick, pale yellow or colorless and transparent. ***Cheilocystidia*** 38–62–89 × 15–21–25 μm (n = 30), mostly similar to pleurocystidia, fusiform to elongate-fusiform, walls pale yellow or colorless. ***Cheiloparacystidia*** 14.8–18.2 × 8.8–11.8 μm, abundant, pyriform or broadly clavate, clustered, thin-walled, colorless, transparent, without distinctive contents. ***Caulocystidia*** 21–68 × 12–30 μm, extending from upper stipe to base, subfusiform or subcylindrical, occasionally utriform or guttiform, apex obtuse, smooth, without crystals, mostly with walls gradually thickening from base to apex, colorless or pale yellow. ***Cauloparacystidia*** 14–34 × 6–17.5 μm, abundant, subglobose, ellipsoid or clavate, thin-walled, colorless, transparent. ***Hymenophoral trama*** 152–185 μm wide, subregular, composed of inflated hyphae 12–27 μm wide, thin-walled, smooth, colorless, some slightly rough. ***Pileipellis*** a cutis, 49–117 μm thick, yellowish-brown to dark brown, composed of elongate cylindrical hyphae, occasionally uplifted, 3.2–5.5 μm wide, thin-walled, surface slightly rough, pale yellow, margin with or without transparent striae. ***Pileal trama*** subregular, composed of inflated hyphae 10–22 μm diam., smooth, thin-walled, transparent, colorless. ***Stipitipellis*** regularly arranged, hyphae cylindrical, smooth, pale yellow, 2.5–5.8 μm wide, mostly smooth, some slightly rough, surface minutely punctate. ***Stipe trama*** colorless, elongate-cylindrical, regularly arranged, 5.0–10.2 μm wide, thin-walled, smooth, colorless. ***Oleiferous hyphae*** 2.5–5.5 μm wide, common in hymenophoral trama and pileipellis, less frequent in pileal trama, branched, with tuberculate nodules, pale yellow to bright yellow. ***Clamp connections*** present in all tissues.

**Figure 9.**
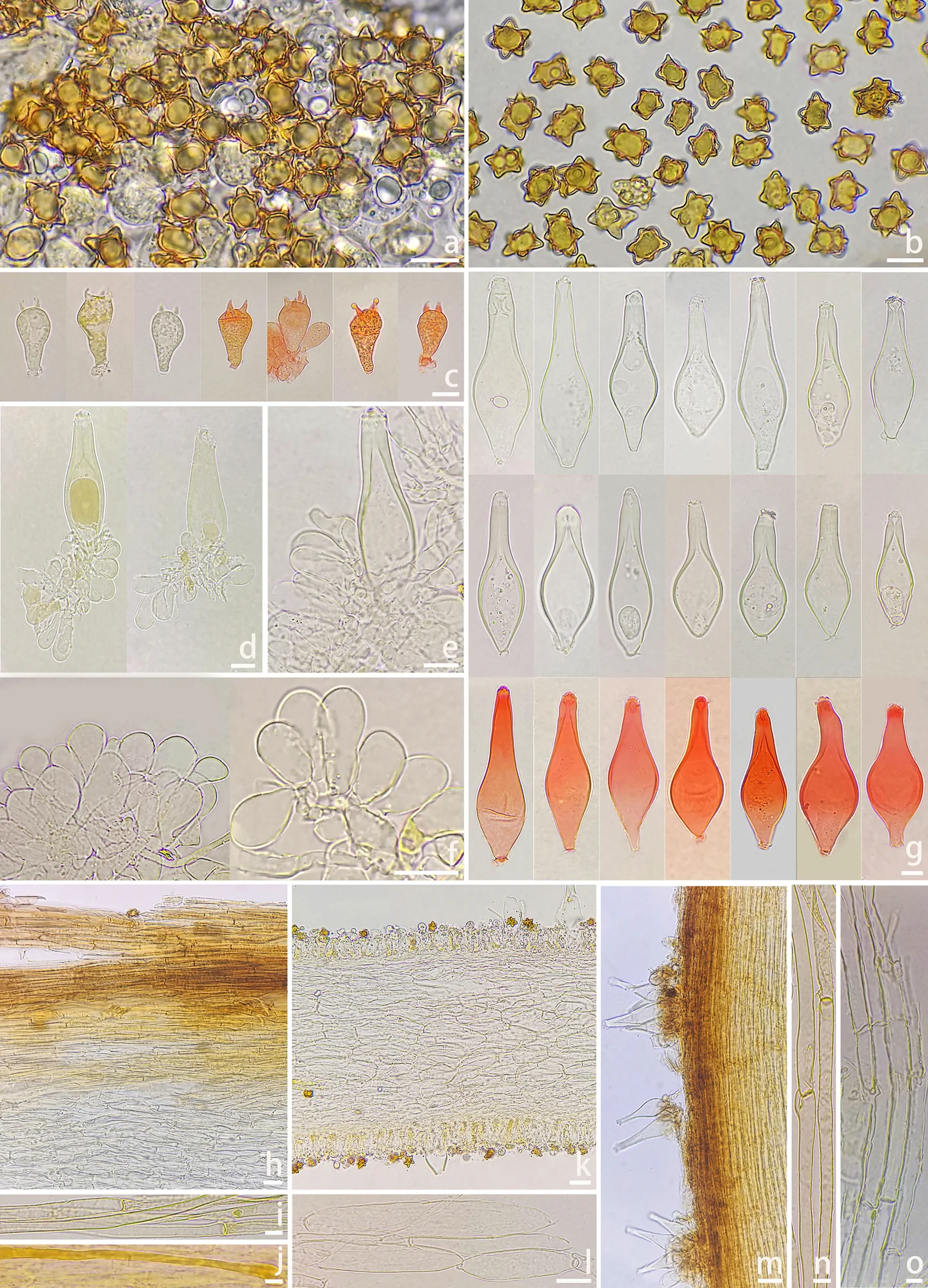
Microscopic features of *Inocybe pretiosa* (FCAS4247, holotype). **a, b**. Basidiospores; **c**. Basidia; **d–f**. Cheilocystidia and cheiloparacystidia; **g**. Pleurocystidia; **h**. Pileipellis and part of the pileal trama; **i**. Pileipellis hyphae; **j**. Oleiferous hyphae; **k**. Cross section of lamellae; **l**. Pileal trama hyphae; **m**. Caulocystidia on stipe surface; **n**. Stipitipellis hyphae; **o**. Stipe trama hyphae. Scale bars: 10 μm (**a–d, f, g, k**); 20 μm (**e, h, i, m**); 5 μm (**j, l, n, o**). Photos by W.Y. Ren.

##### Habitat.

Solitary under broad-leaved forests of *Corylus chinensis*, *Carpinus cordata*, and *Quercus liaotungensis*.

##### Geographical distribution.

Known only from Shaanxi Province, China.

##### Additional specimens examined.

China • Shaanxi Province: Baoji City, Mei County, Tangyu Town, Tangyukou, Taibai Mountain National Forest Park, 34°06'13"N, 107°54'06"E, al. 1342 m, under broad-leaved forests of *Corylus chinensis*, *Carpinus cordata*, *Quercus liaotungensis*, 19 August 2024, leg. Y.G. Fan & J.R. Wang, FYG11183 (FCAS4249), FYG11186 (FCAS4248).

##### Remarks.

This species occurs in the warm-temperate zone of Taibai Mountain National Forest Park, Shaanxi Province, China. It is characterised by relatively small basidiomata with a radially cracked, yellowish-brown to dark brown pileus, a pruinose stipe with a marginately bulbous base, stellate basidiospores with angular nodules, and large utriform hymenial cystidia. The species described from Queensland, *I. nobilissima*, resembles it in the slender, entirely pruinose stipe and spore outline (9.0–12.0 × 8.0–10.0 μm), but *I. nobilissima* has a predominantly yellowish-brown pileus, a buff to brownish stipe, and smaller hymenial cystidia (47–61 × 14–18 μm) (Matheny and Bougher 2017). Phylogenetically, *I. pretiosa* is most closely related to *I. draconoculus*, but the new species clearly differs in having smaller basidiomata, darker pileus color, fewer spore nodules, and markedly larger hymenial cystidia.

## Discussion

In this study, we conducted the first multi-locus phylogenetic assessment of the *I. asterospora* group from warm-temperate and subtropical China, based on ITS, LSU and *rpb2* sequences. Our analyses uncovered three new species, *I. taishanensis*, *I. draconoculus* and *I. pretiosa*, substantially expanding the known species diversity of this group in East Asia. We further demonstrated that previous Chinese records identified as “*I. asterospora*” represent a species complex, and that the confirmed distribution of true *I. asterospora* is currently confined to northern China. Phylogenetically, the *I. asterospora* group shows no clear and stable affiliation with other lineages within sect. *Marginatae* (Fig. 1), which is largely consistent with previous attempts (Aïgnon et al. 2022; Chen et al. 2024; Gao et al. 2025). Collectively, these findings highlight the prevalence of cryptic diversity within the *I. asterospora* group in China, a phenomenon also observed in several wood-inhabiting fungal group (Yuan et al. 2021; Zhou et al. 2023; Song et al. 2025; Wang et al. 2024).

Our phylogenetic framework also allows a critical reassessment of previous global analyses. The topology presented herein differs considerably from that generated by Kuo and Matheny (2015), in which *I. insignis* was recovered as an independent lineage sister to a clade comprising the Asian and Australasian taxa. In our study, however, the tropical Asian clade, the Australasian clade, the *I. insignis* lineage, and the north temperate clade each form separate, well-supported branches. These discrepancies likely stem from differences in sampling strategies and analytical objectives: Kuo and Matheny (2015) focused primarily on the rare North American *I. insignis* with limited representatives from other regions, whereas our study incorporated broader sampling across East Asia, including newly discovered taxa from China. Although the relationships among the four geographic subclades still lack statistical support (Fig. 1), their distinct and non-overlapping distributions strongly point to a history of allopatric diversification. Additional taxon sampling and more molecular markers, or alternative phylogenomic approaches, are needed to further elucidate the origin and evolutionary history of this group.

In addition, our results necessitate a reevaluation of the geographical range of *I. asterospora* sensu stricto. Notably, true *I. asterospora* has a predominantly northern distribution in China, but BLAST searches of ITS sequences extend its range southwards to the Qinling Mountains in Shaanxi Province (KR149754). However, most southern Chinese records lack molecular confirmation and may represent misidentifications or undescribed taxa. A similar situation occurs in North America, where morphological records indicate the presence of *I. asterospora* (Kauffman 1918, 1924), yet no matching sequences exist in GenBank; phylogenetically allied sequences from this region represent at least two undescribed taxa (Kuo and Matheny 2015).

In Europe, three root-tip sequences in GenBank record the association of *I. asterospora* with *Carya* spp. (ON129106, MG835422, and MH834486), a tree genus native to North America but widely introduced across Eurasia. However, studies on ectomycorrhizal communities associated with introduced North American trees in Europe have consistently shown that these exotic hosts predominantly associate with native European fungi, forming novel associations rather than co-introducing their original symbionts from the native range (Wilgan et al. 2020; Wilgan and Leski 2022). Thus, the European records of *I. asterospora* on *Carya* likely reflect a novel association formed by a native European fungus with an introduced host. Consequently, these European sequences cannot be taken as direct evidence supporting the presence of *I. asterospora* in North America. Confirmation of its North American distribution still requires independent molecular evidence obtained from specimens collected within that region.

To date, the *I. asterospora* group comprises ten formally described species, together with several phylogenetic lineages awaiting formal description. Members share a suite of unifying morphological features: a brown to dark brown pileus with conspicuous radial streaks, a brownish to pinkish stipe with a distinctly marginate base, moderately crowded lamellae, stellate (or oblong-angular) basidiospores, thick-walled lageniform hymenial cystidia, thick-walled hymenophoral trama hyphae, and a distinct spermatic odor. Putative hosts include *Fagaceae*, *Betulaceae*, *Juglandaceae*, *Dipterocarpaceae*, *Myrtaceae*, *Nothofagaceae*, and *Fabaceae*. Geographically, the group is distributed across Europe, temperate and tropical Asia, Australia, and North America, with the highest diversity currently documented in East Asia. A global key to species of the *I. asterospora* group was provided as an identification tool (Suppl. material 2).

Since *I. asterospora* produces both muscarine and psilocybin, accurate species identification is critical for tracing poisoning sources and guiding clinical management. Our findings reveal cryptic diversity and a geographically restricted distribution of true *I. asterospora*. Whether the newly discovered species also produce these neurotoxins remains unknown. Toxin screening is therefore necessary to support poisoning prevention and clinical treatment.

## Supplementary Material

XML Treatment for Inocybe asterospora

XML Treatment for Inocybe taishanensis

XML Treatment for Inocybe draconoculus

XML Treatment for Inocybe pretiosa

## Additional information

### Artificial Intelligence (AI) use

The authors accept full responsibility for the content of the manuscript, including the disclosure of any use of AI.

Regarding the use of AI in the preparation of this manuscript, the authors declare the following: DeepSeek.

**Used for**: Language, style and writing. Artificial intelligence tools (Deepseek, https://www.deepseek.com) were only used for basic grammatical checking.

### Author ORCIDs

W.-J. Yu https://orcid.org/0000-0002-0147-3091

Y.-P. Ge https://orcid.org/0000-0001-5754-201X

W.-Y. Ren https://orcid.org/0009-0006-3902-6226

Q. Na https://orcid.org/0000-0001-8406-6389

Y.-R. Xu https://orcid.org/0009-0004-8382-1636

Y.-P. Hu https://orcid.org/0000-0003-1242-1139

Y.-G. Fan https://orcid.org/0000-0002-3028-9752

### Data availability

All of the data that support the findings of this study are available in the main text or Supplementary Information.

## Supplementary materials

Supplementary material 1 Taxon, sequences, and their information Data type pdf Explanation note **table S1**. Taxon, sequences, and their information used in the phylogenetic analyses. https://binary.pensoft.net/file/1802888This dataset is made available under the Open Database License (http://opendatacommons.org/licenses/odbl/1.0/). The Open Database License (ODbL) is a license agreement intended to allow users to freely share, modify, and use this Dataset while maintaining this same freedom for others, provided that the original source and author(s) are credited. Wen-Jie Yu, Yu-Peng Ge, Wan-Yi Ren, Qin Na, Yan-Ru Xu, Ya-Ping Hu, Yu-Guang Fan

Supplementary material 2 A global key to species of the *Inocybe asterospora* group Data type docx https://binary.pensoft.net/file/1802889This dataset is made available under the Open Database License (http://opendatacommons.org/licenses/odbl/1.0/). The Open Database License (ODbL) is a license agreement intended to allow users to freely share, modify, and use this Dataset while maintaining this same freedom for others, provided that the original source and author(s) are credited. Wen-Jie Yu, Yu-Peng Ge, Wan-Yi Ren, Qin Na, Yan-Ru Xu, Ya-Ping Hu, Yu-Guang Fan

## References

[B1] Aïgnon HL, Yorou NS, Ryberg M (2021) State of knowledge on the diversity, phylogeny and distribution of *Inocybaceae* in Africa. Asian Journal of Mycology 4(2): 56–66. 10.5943/ajom/4/2/6

[B2] Aïgnon HL, Jabeen S, Verbeken A, Matheny PB, Yorou NS, Ryberg M (2022) Four new nodulose-spored species of *Inocybe (Agaricales)* from West Africa. Fungal Systematics and Evolution 10(1): 1–18. 10.3114/fuse.2022.10.01 PMC9903347 36789280

[B3] Bandini D, Brandrud TE, Dima B, Dondl M, Fachada V, Hussong A, Mifsud S, Oertel B, Rodriguez Campo FJ, Thos H, Vauras J, Weholt Ø, Eberhardt U (2022) Fibre caps across Europe: type studies and 11 new species of *Inocybe* (*Agaricales*, *Basidiomycota*). Integrative Systematics 5(2): 1–85. 10.18476/2022.901982

[B4] Bau T (2022) Flora Fungorum Sinicorum, Vol. 53, *Inocybaceae*. Science Press, Beijing.

[B5] Breitenbach J, Kränzlin F (2000) *Fungi* of Switzerland, vol. 5, Agarics Part 3, *Cortinariaceae*. Verlag Mykologia, Lucerne.

[B6] Chen JL, Zhang SX, Chen BZ, Zhang HY (1987) Investigation report on poisoning caused by accidental ingestion of *Inocybe asterospora*. Journal of Army Hygiene 5: 36–38.

[B7] Chen X, Yu W-J, Bau T, Matheny PB, Horak E, Liu Y, Qin L-W, Tang L-P, Ge Y-P, Liu T-Z, Fan Y-G (2024) Contributions to the *Inocybe umbratica–paludinella* (*Agaricales*) group in China: Taxonomy, species diversity, and molecular phylogeny. Journal of Fungi 10(10): 893. 10.3390/jof10120893 PMC11677058 39728389

[B8] Dovana F, Bandini D, Eberhardt U, Olariaga I, Bizio E, Ferisin G, Esteve-Raventós F (2023) Re-evaluation of the taxonomic status of species within the *Inocybe similis* complex. Journal of Fungi 9(6): 679. 10.3390/jof9060679 PMC10304480 37367615

[B9] Du F, Liu B, Li ZY (1980) Investigation report on edible and poisonous wild mushrooms in Shanxi (Part 2). Journal of Shanxi University (Natural Science Edition) 3: 71–78.

[B10] Esteve-Raventós F, Bandini D, Oertel B, González V, Moreno G, Olariaga I (2018) Advances in the knowledge of the *Inocybe mixtilis* group (*Inocybaceae*, *Agaricomycetes*), through molecular and morphological studies. Persoonia 41: 213–236. 10.3767/persoonia.2018.41.11 PMC6344818 30728606

[B11] Esteve-Raventós F, Larsson E, Pancorbo F, Scholler M (2022) Typification of *Inocybe oblectabilis* f. *macrospora*, and additional data on the phylogeny of the *Tiliae* and *Pseudohiulca* groups in Europe. Boletín de la Sociedad Micológica de Madrid 46: 49–63.

[B12] Fan YG, Bau T (2010) A revised checklist of the genus Inocybe in China. Journal of Fungal Research 8(3): 189–193.

[B13] Ferrari E (2006) *Inocybe* alpine e subalpine. In: Il Genere *Inocybe* (Fr.) Fr. nel Nord Italia e Paesi Limitrofi. Edizioni Candusso, Alassio.

[B14] Franchi P, Marchetti M, Papetti C (2016) *Inocybe tiliae*, una nuova specie della Sezione *Marginatae*. Rivista di Micologia 59(2): 99–121.

[B15] Gao J-L, Ge Y-P, Matheny PB, He P-M, Wu X-P, Bau T, Yu W-J, Fan Y-G (2025) A phylogeny of the *Inocybe alienospora* group (*Agaricales*) with emphasis on seven new species from China and emendation of sect. *Leptocybe*. Mycology 16(2): 812–855. 10.1080/21501203.2024.2380069 PMC12096670 40415917

[B16] Gardes M, Bruns TD (1993) ITS primers with enhanced specificity for basidiomycetes—application to the identification of mycorrhizae and rusts. Molecular Ecology 2(2): 113–118. 10.1111/j.1365-294X.1993.tb00005.x 8180733

[B17] Ge Y, Liu Z, Zeng H, Cheng X, Na Q (2021) Updated description of *Atheniella* (*Mycenaceae*, *Agaricales*), including three new species with brightly coloured pilei from Yunnan Province, southwest China. MycoKeys 81: 139–164. 10.3897/mycokeys.81.67773 PMC8295246 34305422

[B18] Hall TA (1999) BioEdit: a user-friendly biological sequence alignment editor and analysis program for Windows 95/98/NT. Nucleic Acids Symposium Series 41: 95–98.

[B19] He PM, Fan YG, Deng LS, Yu WJ (2022) *Inocybe carpinicola* (*Inocybaceae*, *Agaricales*), a new nodulose-spored species from Hainan Province, China. Phytotaxa 575(1): 79–88. 10.11646/phytotaxa.575.1.5

[B20] Horak E (1977) Fungi*Agaricini* Novaezelandiae. VI. *Inocybe* (Fr.) Fr. and *Astrosporina* Schroeter. New Zealand Journal of Botany 15(4): 713–747. 10.1080/0028825X.1977.10429642

[B21] Horak E (1979) *Astrosporina (Agaricales)* in Indomalaya and Australasia. Persoonia 10: 157–205.

[B22] Horak E, Matheny PB, Desjardin DE, Soytong K (2015) The genus *Inocybe* (*Inocybaceae*, *Agaricales*, *Basidiomycota*) in Thailand and Malaysia. Phytotaxa 230(3): 201–238. 10.11646/phytotaxa.230.3.1

[B23] Jacobsson S (2008) *Inocybe asterospora*. In: Knudsen H, Vesterholt J (Eds) Funga Nordica, 2^nd^ Edn. Nordsvamp, Copenhagen, 888–889.

[B24] Katoh K, Rozewicki J, Yamada KD (2019) MAFFT online service: multiple sequence alignment, interactive sequence choice and visualization. Briefings in Bioinformatics 20(4): 1160–1166. 10.1093/bib/bbx108 PMC6781576 28968734

[B25] Kauffman CH (1918) The *Agaricaceae* of Michigan. Michigan Geological and Biological Survey, Publication 26, Biological Series 5. W.H. Crawford Co., State Printers, Lansing. 2 vols. 10.5962/bhl.title.58545

[B26] Kauffman CH (1924) *Inocybe* (Fries) Quél. North American Flora 10: 227–260.

[B27] Kobayashi T (2002) The taxonomic studies of the genus *Inocybe*. Nova Hedwigia Beiheft 124. J. Cramer, Berlin, 246 pp.

[B28] Kornerup A, Wanscher JH (1978) Methuen Handbook of Colour. 3^rd^ Edn. Eyre Methuen, London, 252 pp.

[B29] Kuo M, Matheny PB (2015) Contemporary documentation of the rare eastern North American species *Inocybe insignis* (*Inocybaceae*, *Agaricales*). MycoKeys 11: 23–31. 10.3897/mycokeys.11.5604

[B30] Kuyper TW (1986) A revision of the genus *Inocybe* in Europe I. Subgenus *Inosperma* and the smooth-spored species of subgenus *Inocybe*. Persoonia [Supplement] 3: 1–247.

[B31] Larsson E, Vauras J, Cripps CL (2018) *Inocybe praetervisa* group – A clade of four closely related species with partly different geographical distribution ranges in Europe. Mycoscience 59(4): 277–287. 10.1016/j.myc.2017.11.002

[B32] Latha KPD, Manimohan P (2017) Inocybes of Kerala. SporePrint Books, India.

[B33] Li CH, Qu MQ, Yu RL, Zhou CY, Song XX, Wang H, Chen MJ (2016) A preliminary report on species diversity of wild macrofungi in Shanghai. Acta Agriculturae Shanghai 32(4): 59–65.

[B34] Li HJ, Zhang HS, Zhang YZ, Zhang KP, Zhou J, Yin Y, Jiang S, Ma PB, He Q, Zhang Y, Wen K, Yuan Y, Lang N, Lu JJ, Sun CY (2020) Mushroom poisoning outbreaks—China, 2019. China CDC Weekly 2(2): 19–24. 10.46234/ccdcw2020.005 PMC8392910 34594654

[B35] Li HJ, Zhang HS, Zhang YZ, Zhou J, Yin Y, He Q, Jiang S, Ma PB, Zhang Y, Wen K, Yuan Y, Lang N, Cheng B, Lu JJ, Sun CY (2021a) Mushroom poisoning outbreaks—China, 2020. China CDC Weekly 3(3): 41–45. 10.46234/ccdcw2021.014 PMC8392932 34594953

[B36] Li HJ, Zhang HS, Zhang YZ, Zhou J, Yin Y, He Q, Jiang S, Ma PB, Zhang Y, Wen K, Yuan Y, Lang N, Cheng B, Wang M, Sun CY (2022) Mushroom poisoning outbreaks—China, 2021. China CDC Weekly 4(3): 35–40. 10.46234/ccdcw2021.014 PMC8796718 35586461

[B37] Li HJ, Zhang YZ, Zhang HS, Zhou J, Liang JQ, Yin Y, He Q, Jiang S, Zhang Y, Yuan Y, Lang N, Cheng B, Zhong JJ, Li ZF, Sun CY (2023) Mushroom poisoning outbreaks—China, 2022. China CDC Weekly 5(3): 45–50. 10.46234/ccdcw2023.009 PMC9902756 36776462

[B38] Li HJ, Zhang YZ, Zhang HS, Zhou J, Chen ZH, Liang JQ, Yin Y, He Q, Jiang S, Zhang Y, Yuan Y, Lang N, Cheng B, Zhong JJ, Li ZF, Sun CY (2024) Mushroom poisoning outbreaks—China, 2023. China CDC Weekly 6(3): 64–68. 10.46234/ccdcw2024.014 PMC10832152 38313818

[B39] Li HJ, Zhang YZ, Zhang HS, Zhou J, Li ZF, Yin Y, He Q, Jiang S, Zhang Y, Yuan Y, Lang N, Cheng B, Zhong JJ, Yuan M, Liu Z, Sun CY (2025) Mushroom poisoning outbreaks—China, 2024. China CDC Weekly 7(19): 645–649. 10.46234/ccdcw2025.106 PMC12075492 40376178

[B40] Li SN, Xu F, Jiang M, Liu F, Wu F, Zhang P, Fan YG, Chen ZH (2021b) Two new toxic yellow *Inocybe* species from China: morphological characteristics, phylogenetic analyses and toxin detection. MycoKeys 81: 185–204. 10.3897/mycokeys.81.68485 PMC8355026 34385885

[B41] Liang JQ, Chen X, Liu TZ, Zhang YZ, Sun CY, Li ZF, Zhong JJ, Li HJ, Fan YG, Xu F (2026) Mixed toxin profiles in two new and one known species of *Inocybe (Inocybaceae)* from northern China. MycoKeys 141: 23–44. 10.3897/mycokeys.141.203741 PMC13643354 42846760

[B42] Liu Z, Na Q, Cheng X, Wu X, Ge Y (2021) *Mycena yuezhuoi* sp. nov. (*Mycenaceae*, *Agaricales*), a purple species from the peninsula areas of China. Phytotaxa 511(2): 148–162. 10.11646/phytotaxa.511.2.3

[B43] Ludwig E (2017) Pilzkompendium. Vol. 4. Fungicon Verlag, Berlin.

[B44] Lurie Y, Wasser SP, Taha M, Shehade H, Nijim J, Hoffmann Y, Basis F, Vardi M, Lavon O, Suaed S, Bisharat B, Bentur Y (2009) Mushroom poisoning from species of genus *Inocybe* (fiber head mushroom): a case series with exact species identification. Clinical Toxicology 47(6): 562–565. 10.1080/15563650903008448 19566380

[B45] Matheny PB (2005) Improving phylogenetic inference of mushrooms with rpb1 and rpb2 nucleotide sequences (*Inocybe*; *Agaricales*). Molecular Phylogenetics and Evolution 35(1): 1–20. 10.1016/j.ympev.2004.11.014 15737578

[B46] Matheny PB (2009) A phylogenetic classification of the *Inocybaceae*. McIlvainea 18: 11–21.

[B47] Matheny PB, Bougher NL (2017) *Fungi* of Australia: *Inocybaceae*. CSIRO Publishing, Melbourne.

[B48] Matheny PB, Hobbs AM, Esteve-Raventós F (2020) Genera of *Inocybaceae*: New skin for the old ceremony. Mycologia 112(1): 83–120. 10.1080/00275514.2019.1668906 31846596

[B49] Matheny PB, Corrales A, Graddy MG, Halling RE, Kalichman J, Kudzma LV, Ovrebo CL, Swenie RA, Walker NC (2022) A revision of the *Inocybe grammata* group in North America including four new taxa. Brittonia 74(4): 436–464. 10.1007/s12228-022-09720-0

[B50] Nylander JAA (2004) MrModeltest v2.3. Program distributed by the author, Evolutionary Biology Centre, Uppsala University.

[B51] Quélet L (1879) Diagnoses nouvelles de quelques espèces critiques de Champignons. Bulletin de la Société Botanique de France 26: 45–54. 10.1080/00378941.1879.10825724

[B52] Rea C (1922) British *Basidiomycetae*: a handbook to the larger British fungi. Cambridge University Press, London. 10.5962/bhl.title.46431

[B53] Ren J, Wang K, Niu CY, Kang X, Li SM, Wang LA, Liu TZ, Wei TZ, Li GJ (2024) Species diversity and floristic composition of macrofungi in the Yanshan Mountains. Mycosystema 43(11): 240184. 10.13346/j.mycosystema.240184

[B54] Ronquist F, Teslenko M, van der Mark P, Ayres DL, Darling A, Höhna S, Larget B, Liu L, Suchard MA, Huelsenbeck JP (2012) MrBayes 3.2: efficient Bayesian phylogenetic inference and model choice across a large model space. Systematic Biology 61: 539–542. 10.1093/sysbio/sys029 PMC3329765 22357727

[B55] Smith AH (1941) New and unusual Agarics from North America II. Mycologia 33: 1–16. 10.1080/00275514.1941.12020786

[B56] Song CG, Xu TM, Xu YH, Wang D, Zeng L, Fan XP, Sun YF, Cui BK (2025) Systematic revision, molecular phylogeny and divergence times of *Thelephorales (Basidiomycota)*. Mycosphere 16(1): 296–422. 10.5943/mycosphere/16/1/5

[B57] Stangl J (1989) Die Gattung *Inocybe* in Bayern. Hoppea 46: 5–388.

[B58] Tamura K, Peterson D, Peterson N, Stecher G, Nei M, Kumar S (2011) MEGA5: molecular evolutionary genetics analysis using maximum likelihood, evolutionary distance, and maximum parsimony methods. Molecular Biology and Evolution 28(10): 2731–2739. 10.1093/molbev/msr121 PMC3203626 21546353

[B59] Trifinopoulos J, Nguyen LT, Von Haeseler A, Minh BQ (2016) W-IQ-TREE: a fast online phylogenetic tool for maximum likelihood analysis. Nucleic Acids Research 44(W1): 232–235. 10.1093/nar/gkw256 PMC4987875 27084950

[B60] Vilgalys R, Hester M (1990) Rapid genetic identification and mapping of enzymatically amplified ribosomal DNA from several *Cryptococcus* species. Journal of Bacteriology 172(8): 4238–4246. 10.1128/jb.172.8.4238-4246.1990 PMC213247 2376561

[B61] Wang CG, Dai YC, Kout J, Gates GM, Liu HG, Yuan Y, Vlasák J (2024) Multi–gene phylogeny and taxonomy of *Physisporinus* (*Polyporales*, *Basidiomycota*). Mycosphere 15(1): 1455–1521. 10.5943/mycosphere/15/1/12

[B62] Wang JR, Bau T (2009) Species diversity of macrofungi in Jingyuetan National Forest Park. Journal of Jilin Agricultural University 31: 148–156.

[B63] White TJ, Bruns T, Lee S, Taylor J (1990) Amplification and direct sequencing of fungal ribosomal RNA genes for phylogenetics. In: Innis MA, Gelfand DH, Sninsky JJ, White TJ (Eds) PCR Protocols: A Guide to Methods and Applications. Academic Press, San Diego, 315–322. 10.1016/B978-0-12-372180-8.50042-1

[B64] Wilgan R, Leski T (2022) Ectomycorrhizal assemblages of invasive *Quercus rubra* L. and non-invasive *Carya* Nutt. trees under common garden conditions in Europe. Forests 13: 676. 10.3390/f13050676

[B65] Wilgan R, Leski T, Kujawska M, Karliński L, Janowski D, Rudawska M (2020) Ectomycorrhizal fungi of exotic *Carya ovata* in the context of surrounding native forests on Central European sites. Fungal Ecology 44: 100908. 10.1016/j.funeco.2019.100908

[B66] Wu F, Zhou LW, Yang ZL, Bau T, Li TH, Dai YC (2019) Resource diversity of Chinese macrofungi: edible, medicinal and poisonous species. Fungal Diversity 98: 1–76. 10.1007/s13225-019-00432-7

[B67] Xu F, Zhang YZ, Zhang YH, Guan GY, Zhang KP, Li HJ, Wang JJ (2020) Mushroom poisoning from *Inocybe serotina*: A case report from Ningxia, northwest China with exact species identification and muscarine detection. Toxicon 179: 72–75. 10.1016/j.toxicon.2020.03.003 32345453

[B68] Xue JD (1984) Poisonous mushrooms in Henan (II). Journal of Henan Academy of Sciences 3: 40–48.

[B69] Yu WJ, Chang C, Qin LW, Zeng NK, Wang SX, Fan YG (2020) *Pseudosperma citrinostipes* (*Inocybaceae*), a new species associated with *Keteleeria* from southwestern China. Phytotaxa 450(1): 8–16. 10.11646/phytotaxa.450.1.2

[B70] Yuan Y, Chen JJ, Korhonen K, Martin F, Dai YC (2021) An updated global species diversity and phylogeny in the forest pathogenic genus *Heterobasidion* (*Basidiomycota*, *Russulales*). Frontiers in Microbiology 11: 596393. 10.3389/fmicb.2020.596393 PMC7817714 33488542

[B71] Zhou M, Dai YC, Vlasák J, Liu HG, Yuan Y (2023) Updated systematics of *Trichaptum* s.l. (*Hymenochaetales*, *Basidiomycota*). Mycosphere 14: 815–917. 10.5943/mycosphere/14/1/11

[B72] Zhu A-H, Xu Y-R, Ma H-X, Yu W-J (2025) Three nodulose-spored *Inocybe* (*Agaricales*, *Basidiomycota*) species discovered from Motuo, Southwest China. MycoKeys 122: 99–121. 10.3897/mycokeys.122.163942 PMC12447083 40980477

