# Supplementary material for: Phylogeny of the Inocybe asterospora group (Inocybaceae, Agaricales) uncovers cryptic diversity in warm-temperate and subtropical China
Source: MycoKeys. 2026 Oct 6;141:167–94. doi: 10.3897/mycokeys.141.210930 (PMC13662618; doi:10.3897/mycokeys.141.210930)
Supplement: Supplementary file 1 [file mycokeys-141-167-s001.pdf]

Table S1. Taxon, sequences, and their information used in the phylogenetic analyses.

Taxon	Collection no. and/or specimen no.	Locality	GenBank acc. no. ITS	GenBank acc. no. LSU	GenBank acc. no. *rpb2*	References
I. aff. *nodulosa*	130181812	USA	OP749452	—	—	Direct Submission
I. aff. *nodulosa*	130181856	USA	OP749451	—	—	Direct Submission
I. aff. *nodulosa*	92055967	USA	OM809214	—	—	Direct Submission
I. aff. *nodulosa*	26964894	USA	OM473635	—	—	Direct Submission
I. aff. *nodulosa*	129487375	USA	OP743422	—	—	Direct Submission
I. albodiscoides	JK189	Washington	ON116980	ON113316	ON221348	Direct Submission
I. asterospora	**FYG2090-2017**	**China**	**PZ785875**	**PZ785677**	**PZ787503**	**This study**
I. asterospora	**FYG7566**	**China**	**PZ785876**	**PZ785678**	—	**This study**
I. asterospora	EL372-19	France	OP001731	—	—	Direct Submission
I. asterospora	EL100-14	Sweden	MN296110	MN296110	—	Direct Submission
I. asterospora	ZRL20152002	China	LT716046	KY418928	KY419008	Zhao et al. (2017)
I. asterospora	F432	Sweden	PQ653274	—	—	Direct Submission
I. avellaneorosea	AH 23468	Spain	ON994217	ON994217	—	Esteve-Raventós et al. (2023)
I. avellaneorosea	A5058255	Spain	ON994218	ON994218	—	Esteve-Raventós et al. (2023)
I. avellaneorosea	AH51879	France	ON994216	—	—	Direct Submission
I. calocephala	PBM 3600	Australia	—	NG057234	KJ756413	Matheny and Bougher (2017)
I. calospora	JFA 12539	Sweden	—	AY038313	AY337365	Matheny et al. (2002)
I. candidipes	BK-24-July-99-7 (paratype)	USA	AY239019	AY239019	AY239029	Direct Submission
I. casuarinoides	FYG8122	China	OR755899	OR759975	OR775204	Gao et al. (2024)
I. cf.*asterospora*	MR00015	Sweden	AM882897	AM882897	—	Ryberg et al. (2008)
I. diabolica	EL 9006	Sweden	FN550896	FN550896	—	Ryberg et al. (2010)
I. draconoculus	**NJ6366 (holotype)**	**China**	**PZ785873**	**PZ785675**	**PZ787501**	**This study**
I. draconoculus	**WJY20250811-13**		**PZ785874**	**PZ785676**	**PZ787502**	**This study**
I. favrei	Bon84095 (type)	France	KY033786	—	—	Larsson et al. (2018)
I. favrei	G J. Favre 122a (lectotype of. *I. taxocystis*)	Switzerland	JN580884	—	—	Kokkonen and Vauras (2012)

*I. favrei_*	EL5706	Sweden	FN550886	FN550886		Ryberg et al. (2010)
I. flavipes	MR00383 (holotype)	Togo	NR184904	MN097889	MW080915	Aïgnon et al. (2022)
I. flavipes	HLA0363	Benin	MT994601	—	—	Direct Submission
I. hopeae	OR 1665	Thailand	—	ON831503	ON553692	Raghoonundon et al. (2023)
I. insignis	MK07101101	USA	—	KP170913	KM245989	Matheny and Bougher (2015)
I. keteleeriicola	FYG2918	China	PP217742	PP230770	PP238449	Chen et al. (2024)
I. lasseri	MCA 1971	Guyana	—	EU569857	EU569856	Matheny et al. (2009)
I. leptospermi	PBM 3080	New Zealand	KP308760	JN974983	KJ811594	Matheny and Bougher (2017)
I. lineata	DED8048	Thailand	—	GQ892958	KM245999	Horak et al. (2015)
I. lineata	DED8019 (holotype)	Thailand	EU56986	EU56986	—	Matheny et al. (2009)
I. lucida	AH-50955	Spain	ON994205	—	—	Direct Submission
I. lucida	AH-25011	Spain	ON994210	ON994210	—	Esteve-Raventós et al. (2023)
I. luxiensis	FYG2857	China	PP217733	PP230771	PP238450	Chen et al. (2024)
I. muthangensis	K(M) 191735	India	NR160440	NG_064381	KY553247	Latha et al. (2016)
I. nobilissima	PERTH 08364818	Australia	KP308791	KP170961	—	Matheny and Bougher (2017)
I. nobilissima	TENN-067004	Australia	KP308789	—	KM406202	Horak et al. (2015)
I. nobilissima	PERTH_08364788	Australia	KP308790	KP170960	KM406203	Matheny and Bougher (2017)
I. nothomixtilis	AH 46558	Spain, Italy	MT384015	—	MH496025	Esteve-Raventós et al. (2018)
I. oblectabilis	JV32005	Italy	ON754370	ON754370	—	Esteve-Raventós et al. (2022)
I. oblectabilis	M:0222614 (epitype)	Italy	KM873365	—	—	Marchetti et al. (2014)
I. occulta	AH 36443	Spain	NR_160564	—	MH496017	Esteve-Raventós et al. (2018)
I. panamica	AC59	Panama	ON116987	ON113322	ON221353	Matheny et al. (2022)
I. phaeosticta	PAM05091310	France	HQ586859	HQ641102	MH577435	Matheny unpublished
I. phaeosticta	PAM05072305	France	HQ586873	HQ641110	MH577436	Matheny unpublished
I. pileosulcata	DED8164(SFSU)	Thailand	GQ892996	GQ892951	KM406217	Horak et al. (2015)
I. pileosulcata	TBGT10742	India	KP308810	KP170979	KM406218	Direct Submission
I. pileosulcata	CAL 1362	India	KY440098	KY549128	—	Latha and Manimohan (2017)

I. pileosulcata	CAL1368	India	KY440099	KY549129	—	Latha and Manimohan (2017)
I. pingala	CAL 1345	India	KY440100	KY549130	—	Latha and Manimohan (2017)
I. pretiosa	**FYG11178 (holotype)**	**China**	**PZ785870**	**PZ785672**	**PZ787498**	**This study**
I. pretiosa	**FYG11183**	**China**	**PZ785871**	**PZ785673**	**PZ787499**	**This study**
I. pretiosa	**FYG11186**	**China**	**PZ785872**	**PZ785674**	**PZ787500**	**This study**
I. rekhankitha	CAL1356	India	KY440102	KY549132	—	Latha and Manimohan (2017)
*I. rivularis_*	JV-3610F_(paratype)	Finland	JN580885	—	—	Direct Submission
I. sepiana	SMNS-STU-F-0901687 (holotype)	Germany	OP164056	OP164056	—	Bandini et al. (2022)
I. sepiana	SMNS-STU-F-0901688	Germany	OP164103	OP164103	—	Bandini et al. (2022)
I. sepiana	SMNS-STU-F-0901705	Germany	OP164104	OP164104	—	Bandini et al. (2022)
I. silvana	CAL1259	India	KY440104	KY549134	—	Direct Submission
***I.* sp.**	**NJ6170**	**China**	**PZ785877**	**PZ785679**	**PZ787504**	**This study**
I. sp.	ASV1281	Hungary	PZ142176	—	—	Geml et al. (2022)
I. sp.	ASV1022	Hungary	OP042650	—	—	Geml et al. (2022)
I. sp.	YM4584	Japan	AB922918	—	—	Miyamoto et al. (2015)
I. sp.	Pj3-mOTU036	Japan	LC260444	—	—	Murata and Nara (2017)
I. tabacina	PAM05071302	France	HQ586865	HQ641106	MH577481	Matheny unpublished
*I. tabacina_*	HMUT M1287	China	—	ON430593	ON470149	Direct Submission
I. taishanensis	**FYG9918 (holotype)**	**China**	**PZ785878**	**PZ785680**	**PZ787505**	**This study**
I. taishanensis	**FYG9935**	**China**	**PZ785879**	**PZ785681**	**PZ787506**	**This study**
I. taishanensis	**FYG12863**	**China**	**PZ785880**	**PZ785682**	**PZ787507**	**This study**
I. tiliae	AMB 18103 (holotype)	Italy	KX592681	—	—	Franchi et al. (2016)
I. tiliae	KR-M-0038132	Germany	OP164036	OP164036	—	Bandini et al. (2022)
I. tiliae	SMNS-STU-F-0900 994	Germany	OP164070	OP164070	—	Bandini et al. (2022)
I. torresiae	PBM 3722	Australia	KP641634	KP171087	KM656131	Matheny and Bougher (2017)
I. tundrae	M0216707 (holotype)	Sweden	MK153627	—	—	Cripps et al. (2020)

I. vagata	PBM3187	Australia	KP641639	KP171091	KM656135	Matheny and Bougher (2017)
I. velata	MCVE 23506 (holotype)	Italy	OP164047	—	—	Bandini et al. (2022)
I. velata	SMNS-STU-090172 1	Germany	OP164085	OP164085	—	Bandini et al. (2022)
I. viscata	TENN-066121	Australia	KP641647	KP171096	MH577490	Matheny and Bougher (2017)
I. xanthomelas	PAM 08082901	France	HQ586856	HQ641097	—	Direct Submission
Nothocybe distincta	ZT 9250	India	—	EU604546	EU600904	Latha et al. (2016)
N. distincta	CAL 1310	India	NR173156	NG057278	KX171345	Latha et al. (2016)
