# Supplementary material for: Phylogeny of the Inocybe asterospora group (Inocybaceae, Agaricales) uncovers cryptic diversity in warm-temperate and subtropical China
Source: MycoKeys. 2026 Oct 6;141:167–94. doi: 10.3897/mycokeys.141.210930 (PMC13662618; doi:10.3897/mycokeys.141.210930)
Supplement: Supplementary file 2 [file mycokeys-141-167-s002.docx]

**A global keye to species of the *Inocybe asterospora* group**

**1.** Basidiomata with greenish blue bruising reaction............................................... ***Inocybe insignis***
**1.** Basidiomata without greenish blue bruising reaction....................................................................2

**2.** Occurring in the Northern Hemisphere..........................................................................................3
**2.** Occurring in the Southern Hemisphere..........................................................................................8

**3.** Distributed in north temperate regions...........................................................................................4
**3.** Distributed in tropical Asia............................................................................................................7

**4.** Pileus and stipe chestnut brown or somewhat paler.......................................................................5
**4.** Pileus and stipe greyish brown to dark brown...............................................................................6

**5.** Basidiospores stellate, hymenial cystidia with colorless walls.................................***I. asterospora***

**5.** Basidiospores oblong with angular projections, hymenial cystidia with yellowish walls.............................................................................................................................***I. taishanensis***

**6.** Pileus dark brown to reddish-brown with appressed fibrils; stipe paler towards apex, basidiospores with less than 11 projections.........................................................................***I. pretiosa***
**6.** Pileus greyish-brown to pale brown with even fine rimulose cracks; stipe uniformly coloured, basidiospores with more than 11 projections.............................................................***I. draconoculus***

**7.** Pileus pale brown with radial streaks; hymenial cystidia fusiform or utriform.........................................................................................................***I. pileosulcata/I. silvana***
**7.** Pileus dark brown with fibrillose squamules; hymenial cystidia broadly fusiform........................................................................................................................***I. rekhankitha***

**8.** Basidiomata has more brownish color, hymenial cystidia fusiform with a length < 61 μm..................................................................................................................................***I. nobilissima***

**8.** Basidiomata pale brown, hymenial cystidia sublageniform with a length > 62 μm..........................................................................................................................................***I. vagata***
